# Z-DNA-binding protein 1 exacerbates myocardial ischemia‒reperfusion injury by inducing noncanonical cardiomyocyte PANoptosis

**DOI:** 10.1038/s41392-025-02430-5

**Published:** 2025-10-07

**Authors:** Xiaokai Zhang, Shuai Song, Zihang Huang, Linqi Zeng, Yining Song, Mohan Li, Chenyan Liu, Fengze Cai, Tongyao Wang, Peng Yu, Junbo Ge, Aijun Sun

**Affiliations:** 1 Department of Cardiology, Zhongshan Hospital, Fudan University, Shanghai Institute of Cardiovascular Diseases, Shanghai, China; 2 State Key Laboratory of Cardiology, Zhongshan Hospital, Fudan University, Shanghai, China; 3 Key Laboratory of Viral Heart Diseases, National Health Commission, Shanghai, China; 4 Key Laboratory of Viral Heart Diseases, Chinese Academy of Medical Sciences, Shanghai, China; 5 National Clinical Research Center for Interventional Medicine, Shanghai, China; 6 Institutes of Biomedical Sciences, Fudan University, Shanghai, China; 7 Qidong-Fudan Innovative Institution of Medical Sciences, Qidong, Jiangsu China

**Keywords:** Cardiology, Cell biology

## Abstract

Myocardial ischemia‒reperfusion (I/R) injury is the primary factor that counteracts the beneficial effects of reperfusion therapy. Cardiomyocyte death serves as the fundamental pathological hallmark of I/R injury. However, targeting a single type of cell death has been reported to be ineffective at preventing I/R injury. ZBP1 is well established as a nucleic acid sensor that activates inflammatory and various cell death signaling pathways. However, the specific role of ZBP1 in adult cardiomyocytes, particularly in the absence of nucleic acid ligands, remains largely unexplored. In this study, our dynamic transcriptomic analyses at various I/R stages revealed a cluster of genes significantly enriched in cell death-related processes, with ZBP1 showing significant expression changes in both our I/R injury mouse model and public human ischemic cardiomyopathy datasets. Cardiomyocytes are the primary cell type expressing ZBP1 in response to I/R injury. Hypoxia/reoxygenation stress induced the upregulation of multiple cell death markers indicative of PANoptosis in adult cardiomyocytes, which was mitigated by ZBP1 deficiency. Compared with treatment with conventional cell death inhibitors, cardiomyocyte-specific Zbp1 deficiency ameliorated I/R-induced PANoptosis, resulting in a more substantial reduction in myocardial infarct size. Conversely, myocardial Zbp1 overexpression in adult mice directly induced cardiac remodeling and heart failure. Mechanistically, ZBP1 drives cardiomyocyte PANoptosis by promoting the formation of the ZBP1/RIPK3/CAS8/CAS6 PANoptosome complex. Virtual screening and experimental validation revealed a novel small-molecule compound, MSB, which has high binding affinity for ZBP1 and effectively attenuates myocardial I/R injury both in vitro and in vivo. Collectively, these findings highlight the role of ZBP1 as a mediator of cardiomyocyte PANoptosis and suggest that targeting ZBP1 could be a promising strategy for mitigating myocardial I/R injury.

## Introduction

Myocardial infarction has emerged as the predominant cause of morbidity and mortality worldwide.^1^ Although standard treatments, such as emergency revascularization, effectively restore the blood supply to ischemic tissues, the reperfusion process can induce additional cardiomyocyte death and sustained cardiac damage. This process is characterized by myocardial ischemia‒reperfusion (I/R) injury, which counteracts the favorable effects of reperfusion therapy.^2^ Consequently, a clinical need remains for elucidating the pathogenic mechanisms underlying I/R injury and developing therapeutic strategies to mitigate its detrimental consequences.^3^

Excessive death of cardiomyocytes is the fundamental pathological hallmark of myocardial I/R injury. The loss of terminally differentiated cardiomyocytes not only increases infarct size during the acute phase but also contributes to long-term cardiac remodeling and the development of heart failure.^4^ Apoptosis and necrosis were initially identified as the primary modes of cell death implicated in myocardial I/R injury. Recently, novel forms of regulated cell death distinct from apoptosis, including necroptosis, pyroptosis, and ferroptosis, have been identified in cardiovascular diseases.^5^ However, the extent and mechanisms of the interactions among the different forms of cell death during I/R injury remain elusive. A series of previous studies have proposed a unified paradigm of a highly coordinated cell death process, PANoptosis, which displays molecular characteristics similar to those of pyroptosis, apoptosis, and necroptosis.^6,7^ PANoptosis is regulated by the PANoptosome complex, which is composed of pivotal molecules from all three cell death pathways. The molecular composition of the PANoptosome is context-dependent, varying according to the initiating stimulus; nevertheless, it typically comprises three principal categories of components: pattern recognition receptors that sense pathogen- or damage-associated molecular patterns (PAMPs or DAMPs), adapter proteins and catalytic effector molecules.^8^ PANoptosis represents a central mechanism of the innate immune response and can be activated by diverse pathogenic stimuli. Apart from infectious diseases, accumulating evidence suggests that PANoptosis is intricately involved in the initiation and progression of diverse pathological conditions, including autoinflammatory diseases and cancer.^9^ Recent research has also revealed the existence of PANoptosis-like cell death in neuronal ischemia, thus expanding our comprehension of the triggers associated with PANoptosis.^10^ Nevertheless, the involvement of PANoptosis and its associated mechanisms in myocardial I/R injury must be further investigated.

Z-DNA-binding protein 1 (ZBP1), also known as DNA-dependent activator of interferon regulatory factors (DAI) or DLM1, was originally identified as the first innate immune sensor of cytoplasmic DNA, particularly double-stranded nucleic acids adopting a Z-conformation.^11,12^ The protein shares 47% sequence homology with its murine counterpart and 46% with the rat ortholog.^13^ Structurally, ZBP1 consists of two N-terminal Z-nucleic acid (Z-NA)-binding domains (Zα1 and Zα2), two central receptor-interacting protein homotypic interaction motifs (RHIMs), and a C-terminal signaling domain (SD).^14^ The Zα domains regulate cytoplasmic distribution, including localization to stress granules (SGs) and processing bodies (PBs), while primarily mediating specific recognition of left-handed double-stranded nucleic acids (Z-DNA and Z-RNA).^15^ The RHIM domains facilitate homotypic RHIM–RHIM interactions, enabling the recruitment of RHIM-containing kinases such as receptor-interacting serine/threonine kinase 1 (RIPK1) and receptor-interacting protein kinase 3 (RIPK3), which initiate inflammation and programmed cell death in a context-dependent manner.^16^ Finally, the SD domain mediates type I interferon signaling in response to immune-stimulatory DNA by interacting with TANK-binding kinase 1 (TBK1) and interferon regulatory factor 3 (IRF3).^17,18^ The structure of ZBP1 enables it to modulate the assembly of a prototypical PANoptosome and initiate PANoptosis. Growing evidence has suggested that various microbial infections can trigger PANoptosis in a ZBP1-dependent manner, serving as a vital component of the innate immune defense by facilitating the elimination of infected cells.^6,19,20^ However, given the unique characteristics of adult cardiomyocytes, such as their limited proliferative capacity and highly organized structure, the specific role of ZBP1 in these cells remains largely unexplored.

In this study, time series RNA sequencing (RNA-seq) analyses revealed a cluster of genes whose expression was upregulated during the reperfusion phase following myocardial ischemia. These genes are associated with cell death-related processes. Further integrated analysis revealed that ZBP1 shows significant expression changes in both our I/R injury mouse model and public human ischemic cardiomyopathy (ICM) datasets. Cardiomyocytes are the primary cell type expressing ZBP1 in response to I/R injury. Hypoxia/reoxygenation stress induced the upregulation of multiple cell death markers indicative of PANoptosis in adult cardiomyocytes, which was mitigated by ZBP1 deficiency. Conditional *Zbp1* deficiency in mouse cardiomyocytes mitigated I/R-induced PANoptosis and cardiac remodeling. Conversely, conditional overexpression of *Zbp1* directly induced the development of cardiac remodeling and heart failure, which could be attenuated by cell death inhibitors. Mechanistically, ZBP1 drives cardiomyocyte PANoptosis by modulating noncanonical ZBP1/RIPK3/CAS8/CAS6 PANoptosome assembly. Subsequently, virtual screening identified a novel small-molecule compound, MSB, which was further validated experimentally to exhibit high binding affinity for ZBP1 and to effectively mitigate myocardial ischemia–reperfusion injury both in vitro and in vivo. Collectively, these findings highlight the role of ZBP1 as a mediator of cardiomyocyte PANoptosis and suggest its potential as a novel therapeutic target for myocardial I/R injury.

## Results

### Integrative bioinformatics analyses identified an essential role of ZBP1 in myocardial I/R injury

**Fig. 1:**
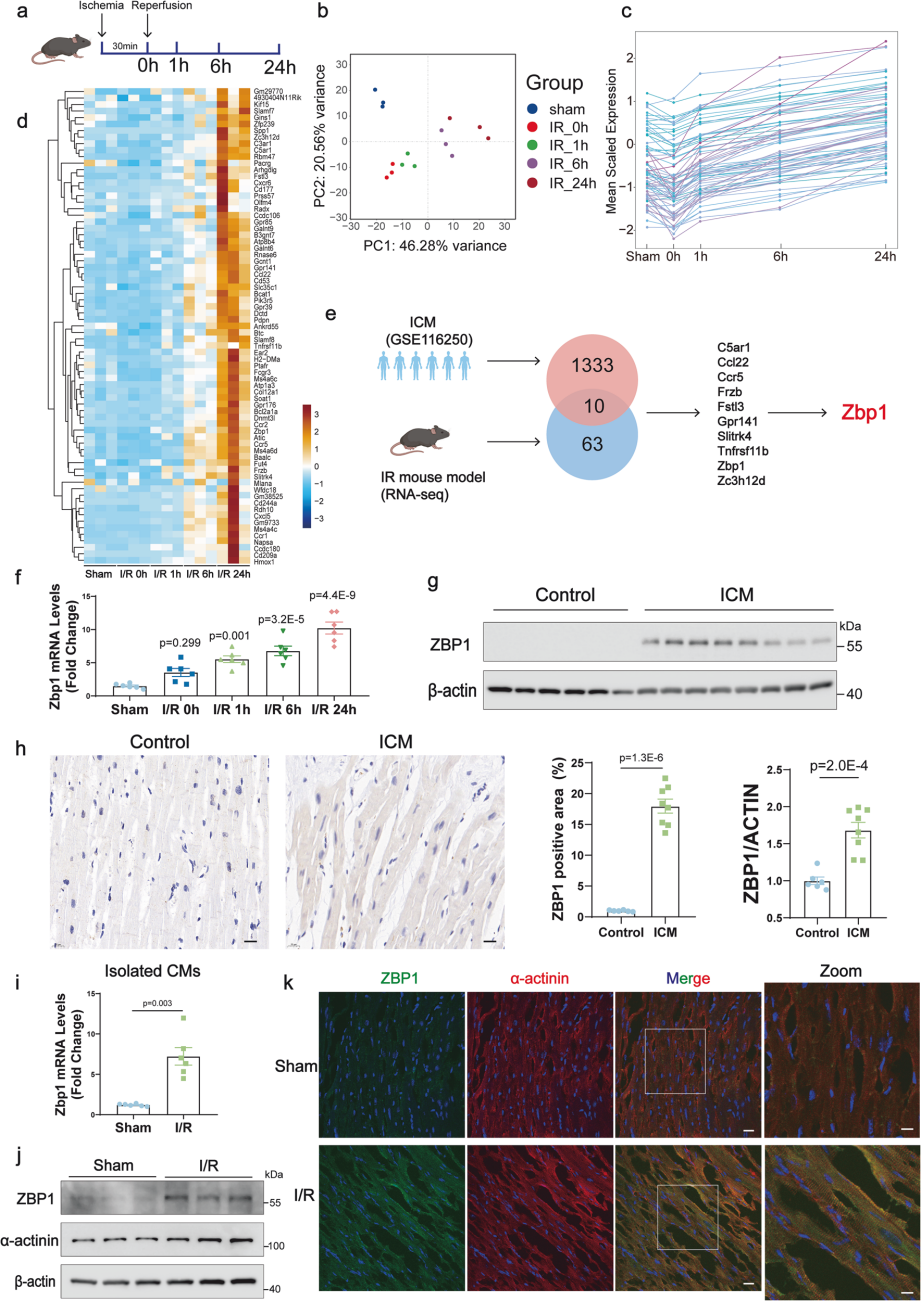
Integrative bioinformatics analyses identified an essential role of ZBP1 in myocardial I/R injury. **a** Schematic diagram showing the mouse model of I/R injury. **b** Principal component analysis (PCA) showing group distribution on the basis of RNA-seq analyses of myocardial samples from the ischemic regions of wild-type (WT) mice subjected to sham surgery or after 30 min of ischemia followed by reperfusion (duration 0, 1, 6, and 24 h). **c** The dynamic change patterns of differentially expressed genes (DEGs) remained unchanged at 0 h post-I/R but were consistently upregulated at 0, 1, 6, and 24 h post-I/R. **d** Heatmap of DEGs that remained unchanged at 0 h post-I/R but were consistently upregulated at 0, 1, 6, and 24 h post-I/R. **e** Schematic illustration of the screening process for candidate genes. **f** Quantitative real-time polymerase chain reaction (qRT‒PCR) analyses of Zbp1 mRNA levels in heart samples from WT mice at 0, 1, 6, and 24 h post-I/R or sham surgery (*n* = 6). **g** Western blot analysis of ZBP1 protein levels normalized to that of β-actin (ZBP1/actin ratio) in heart samples from normal controls and ICM patients (*n* = 6 for normal controls and *n* = 8 for ICM patients). **h** Representative immunohistochemical staining and quantification of ZBP1 in heart samples from normal controls and ICM patients (*n* = 6 for normal controls and *n* = 8 for ICM patients; scale bar = 20 μm). The hematoxylin-stained nuclei appeared blue, while the DAB-positive signal was brown-yellow. **i** Quantitative real-time polymerase chain reaction (qRT‒PCR) analysis of Zbp1 mRNA levels in cardiomyocytes isolated from WT mice 24 h post-I/R or sham surgery (*n* = 6). **j** Western blot of ZBP1 in cardiomyocytes isolated from WT mice 24 h post-I/R or sham surgery (*n* = 3). **k** Immunofluorescence of 4′,6-diamidino-2-phenylindole (DAPI; blue), ZBP1 (green) and α-actinin (red) in the myocardium of WT mice 24 h post-I/R or sham surgery (scale bar = 20 μm in the left panel and 10 μm in the right panel). For all the statistical plots, the data are presented as the means ± SEMs. **f** by one-way ANOVA with Bonferroni multiple comparison test. **g** by two-tailed unpaired Student’s t-test. **h**, **i** by Welch’s t-test

To elucidate the dynamic molecular changes following myocardial I/R injury, time-series RNA-seq analyses were conducted on myocardial samples from the ischemic regions of wild-type (WT) mice after being subjected to either sham surgery or 30 min of ischemia followed by reperfusion (duration: 0, 1, 6, and 24 h) (Fig. 1a). Principal component analysis (PCA) clearly revealed segregation of gene expression profiles between the ischemic and reperfusion stages of myocardial I/R injury (Fig. 1b). To identify genes predominantly affected by reperfusion, a total of 4,436 differentially expressed genes (DEGs, false discovery rate (q value) <0.05; fold change >1.5 or <1/1.5) between the two groups (0 h post-I/R vs. 24 h post-I/R) were categorized into six distinct clusters on the basis of their expression profiles during the ischemic and reperfusion stages. Compared with those in the sham group, genes whose expression increased from 0 to 24 h during reperfusion with different statuses after 30 min of ischemia (0 h of reperfusion) were categorized into clusters 1‒3 (Supplementary Fig. 1). Similarly, genes whose expression decreased from 0 to 24 h during reperfusion with different statuses after 30 min of ischemia compared with that in the sham group were categorized into clusters 4‒6 (Supplementary Fig. 1). Notably, the majority of the DEGs were in cluster 3, and gene ontology (GO) analysis revealed significant enrichment in cell death-related processes. Therefore, cluster 3 was of particular interest, prompting further screening analysis to identify potential candidates involved in regulating I/R injury within this cluster. Among the 1607 DEGs in cluster 3, 73 genes remained unchanged at 0 h post-I/R injury but were consistently upregulated at 1, 6, and 24 h post-I/R injury (Fig. 1c, d). To investigate whether these genes exhibited a consistent expression pattern in human myocardial ischemia, we analyzed a published RNA-seq dataset (GSE116250) and identified 1343 genes upregulated in the left ventricles of patients with ICM. By intersecting these findings with our mouse RNA-seq data, we identified 10 overlapping genes (Fig. 1e). ZBP1 has emerged as a key mediator of cell death that has not been previously reported in the context of myocardial I/R injury. Quantitative real-time polymerase chain reaction (qRT‒PCR) confirmed that the mRNA levels of ZBP1 were consistent with the RNA-seq results (Fig. 1f). Western blot and immunohistochemical analyses further demonstrated increased ZBP1 protein levels in the ICM samples (Fig. 1g, h). Collectively, these findings indicate a potential association between elevated ZBP1 levels and the reperfusion phase of myocardial I/R injury, emphasizing the need for future research into the role of ZBP1 in this process.

### Cardiomyocytes are the primary cell type expressing ZBP1 in response to I/R injury

Next, we investigated the specific cardiac cell populations contributing to the increased expression of ZBP1 in response to myocardial I/R injury. Cardiomyocytes and non-cardiomyocytes were isolated from the ischemic regions of hearts from 8-week-old WT mice subjected to I/R or sham surgery for 24 h. Myocardial *Zbp1* expression was evaluated via qRT‒PCR. Cardiomyocytes presented elevated *Zbp1* expression following I/R (Fig. 1i, j), whereas I/R had no significant effect on *Zbp1* expression in fibroblasts (Supplementary Fig. 2a, b), endothelial cells (Supplementary Fig. 2d, e), or immune cells (Supplementary Fig. 2g, h). Immunofluorescence staining further confirmed a marked increase in ZBP1 within cardiomyocytes (Fig. 1k), with no colocalization observed in fibroblasts, endothelial cells, or immune cells, in cardiac tissues from mice subjected to I/R surgery (Supplementary Fig. 2c, f, i). These findings indicate that cardiomyocytes are primarily responsible for elevated myocardial ZBP1 expression after I/R.

### *Zbp1* deficiency attenuates pyroptosis, apoptosis, and necroptosis in cardiomyocytes after H/R stress

**Fig. 2:**
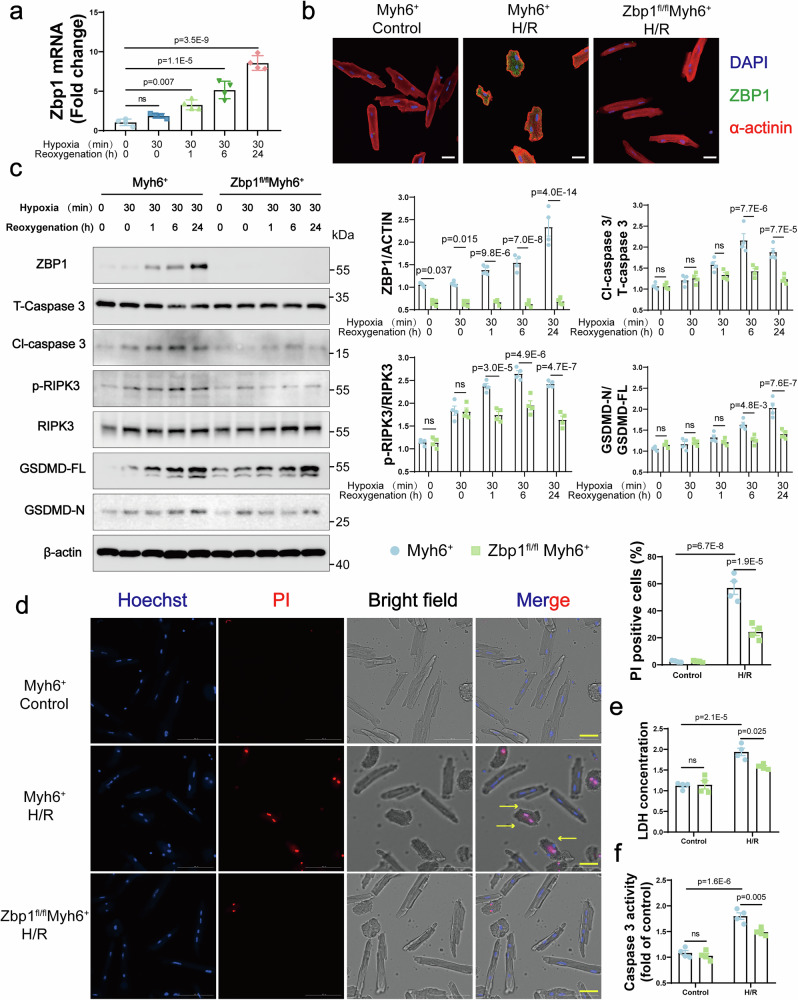
*Zbp1* knockdown attenuates pyroptosis, apoptosis, and necroptosis in cardiomyocytes after H/R stress. **a** Quantitative real-time polymerase chain reaction (qRT‒PCR) analyses of Zbp1 mRNA levels in adolescent cardiomyocytes subjected to H/R for different durations (*n* = 4). **b** Immunofluorescence of ZBP1 in adult *Myh6*^+^ and *Zbp1*^*fl/fl*^*Myh6*^+^ mouse cardiomyocytes subjected to H/R for 0.5/24 h (scale bar = 20 μm). **c** Western blot and quantification of ZBP1, total caspase 3 (T-Caspase 3), cleaved caspase 3 (Cl-Caspase 3), phosphorylated-RIPK3 (p-RIPK3), RIPK3, full-length GSDMD (GSDMD-FL) and GSDMD-N fragment protein levels in adult *Myh6*^+^ and *Zbp1*^*fl/fl*^*Myh6*^+^ mouse cardiomyocytes subjected to H/R for different durations (*n* = 4). **d** Microscopy images and quantification of adult cardiomyocytes induced by H/R for 0.5/24 h were obtained via propidium iodide (PI) and Hoechst staining (*n* = 4; scale bar = 50 μm). **e** LDH (lactate dehydrogenase) release by adult *Myh6*^+^ and *Zbp1*^*fl/fl*^*Myh6*^+^ mouse cardiomyocytes subjected to H/R for 0.5–24 h (*n* = 4). **f** Caspase-3 activity in the cardiomyocytes of adult *Myh6*^+^ and *Zbp1*^*fl/fl*^*Myh6*^+^ mice subjected to H/R for 0.5/24 h (*n* = 4). For all the statistical plots, the data are presented as the means ± SEMs. **a** by one-way ANOVA with Bonferroni multiple comparison test. **c** and **e**, **f** by two-way ANOVA with Bonferroni multiple comparison test. **d** by Welch’s ANOVA with Dunnett’s T3 multiple comparison test

To further investigate the role of ZBP1 in cardiomyocytes, adult cardiomyocytes were isolated and subjected to hypoxia/reoxygenation (H/R) stress as an in vitro model of myocardial I/R injury. Consistent with our in vivo findings, *Zbp1* mRNA levels steadily increased at 1, 6, and 24 h after H/R (Fig. 2a). Immunofluorescence staining further confirmed the significant upregulation of ZBP1 in cardiomyocytes after H/R (Fig. 2b). Next, cardiomyocytes were isolated from global cGAS-deficient (*cGAS*^−/−^) and Sting-deficient (*Sting*^−/−^) mice to investigate whether the cGAS‒STING pathway mediates H/R-induced ZBP1 upregulation. Notably, although depletion of cGAS or STING in adult cardiomyocytes inhibited the upregulation of interferon-stimulated genes (ISGs) such as Ifit1 and Ifi44, ZBP1 expression continued to increase (Supplementary Fig. 3a–d). These findings suggest that the H/R-induced upregulation of ZBP1 is independent of the cGAS‒STING signaling pathway.

Because ZBP1 has been demonstrated to function as a central regulator of programmed cell death, we aimed to investigate whether ZBP1 was responsible for H/R-induced cardiomyocyte death. Therefore, we generated *Zbp1* conditional knockout mice (*Zbp1*^*fl/fl*^*Myh6*^+^) by crossing *Zbp1*-floxed (*Zbp1*^*fl/fl*^) mice with *Myh6*-Cre^ERT2^ mice, enabling the tamoxifen (TAM)-inducible, cardiomyocyte-specific knockout of *Zbp1* (Supplementary Fig. 4a). PCR analysis was used to genotype the mice (Supplementary Fig. 4b). After 5 days of continuous TAM administration (50 mg/kg), the *Zbp1*^*fl/fl*^*Myh6*^+^ and *Myh6*^*+*^ mice had 7 days to acclimate to the effects. Because we used 8-week-old mice, which have significantly lower cardiomyocyte ZBP1 expression than non-cardiomyocytes (Supplementary Fig. 4c), the knockout efficiency was confirmed by evaluating *Zbp1* expression via qRT‒PCR in cardiomyocytes isolated from both the *Zbp1*^*fl/fl*^*Myh6*^+^ and control *Myh6*^*+*^ mice. Indeed, cardiomyocyte-specific *Zbp1* knockout was confirmed in the *Zbp1*^*fl/fl*^*Myh6*^+^ mice (Supplementary Fig. 4d, e). Immunofluorescence staining revealed that approximately 80% of ZBP1 expression was abrogated following TAM administration (Supplementary Fig. 4f). Next, we performed a time series analysis of adult cardiomyocytes after 0.5 h of hypoxia, followed by varying durations of reoxygenation. Western blot analysis revealed that H/R significantly increased the expression of pyroptosis, apoptosis, and necroptosis markers in a time-dependent manner. All of these markers simultaneously increased between 0 and 24 h of reoxygenation, thereby indicating a PANoptosis phenotype. In contrast, ZBP1 deficiency markedly inhibited H/R-induced cardiomyocyte pyroptosis, apoptosis, and necroptosis during the reoxygenation phase (Fig. 2c). Consistently, ZBP1 deficiency also prevented the loss of cardiomyocyte membrane integrity, as indicated by the reduced presence of propidium iodide (PI)-positive cardiomyocytes, balloon-shaped vesicles (yellow arrow) and lactate dehydrogenase (LDH) release (Fig. 2d, e). Cellular caspase 3 (CASP3) activity was also reduced in *Zbp1*^*fl/fl*^*Myh6*^+^ mouse cardiomyocytes (Fig. 2f).

Previous studies have reported the critical role of ZBP1 in mediating inflammation across various cell types,^21^ a process that can be triggered by inflammatory forms of cell death, which in turn may amplify the initiation of further cell death. However, we did not find significant inhibition of H/R-induced activation of cGAS‒STING signaling, IFN-I responses or NFκB signaling in ZBP1-deleted adult cardiomyocytes (Supplementary Fig. 5a–d). Taken together, these findings suggest that H/R stress induces the upregulation of ZBP1 in cardiomyocytes to drive PANoptosis-like cell death independently of inflammatory responses.

### Targeting ZBP1 protects human iPSC-derived cardiomyocytes against H/R stress

To further elucidate the role of the human analog of ZBP1 in the context of I/R injury, human induced pluripotent stem-cell-derived cardiomyocytes (iPSC-CMs) were subjected to H/R stress. qRT‒PCR and immunofluorescence analysis confirmed the significant upregulation of ZBP1 following H/R (Supplementary Fig. 6a, b). Consistent with the findings in rodent cardiomyocytes, ZBP1 knockdown in iPSC-CM mitigated H/R-induced cardiomyocyte death, as indicated by a reduced number of PI-positive cells, decreased LDH release, and lower CASP3 activity (Supplementary Fig. 6c–e). These data further highlight the potential clinical importance of targeting ZBP1 to reduce myocardial I/R injury.

### Cardiomyocyte-specific *Zbp1* deficiency ameliorates I/R-induced cardiomyocyte pyroptosis, apoptosis, and necroptosis in vivo

**Fig. 3:**
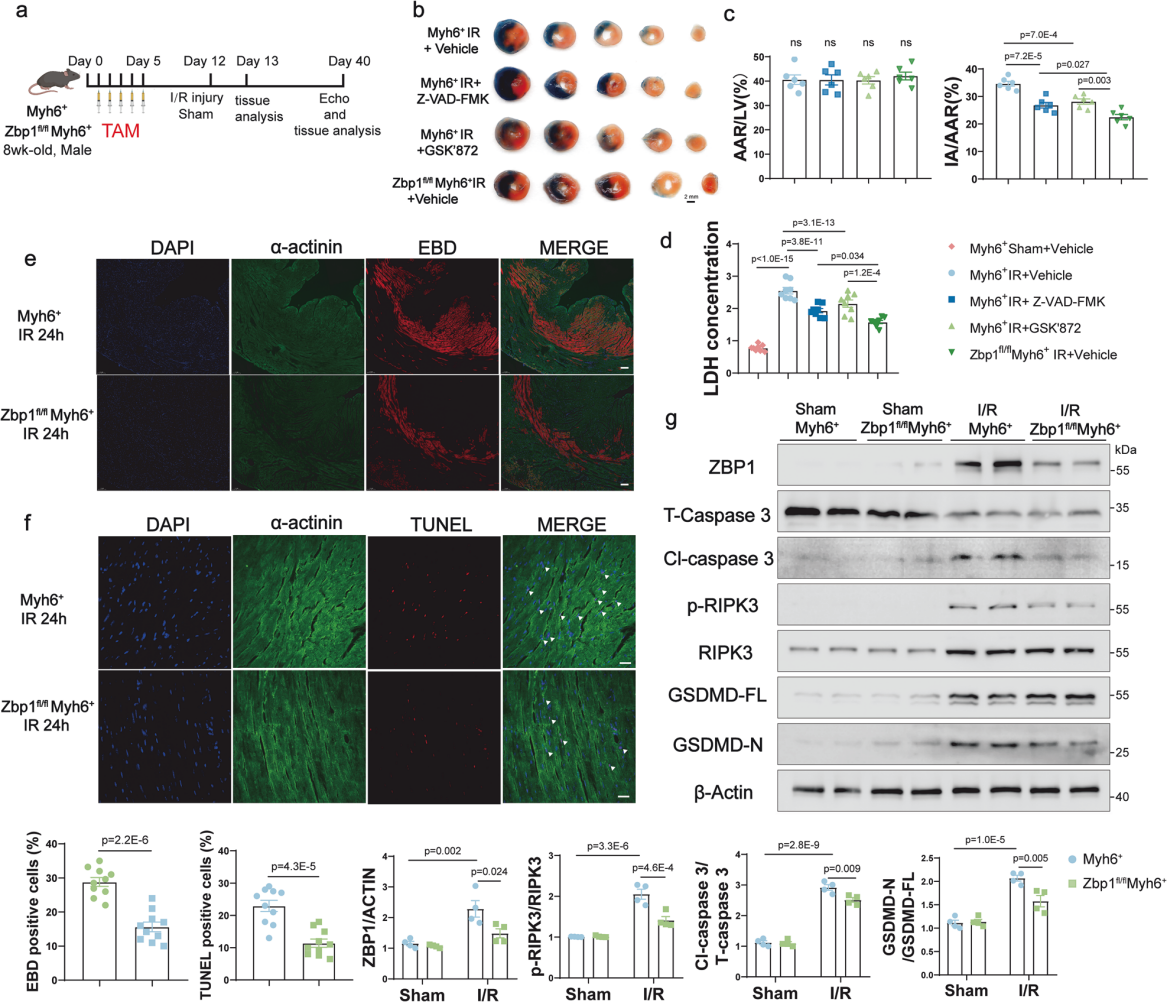
Cardiomyocyte-specific *Zbp1* deficiency ameliorates I/R-induced myocardial injury in vivo. **a** Schematic diagram showing the mouse model of I/R injury. The *Myh6*^+^ and *Zbp1*^*fl/fl*^*Myh6*^+^ mice were intraperitoneally injected with Z-VAD-FMK (20 mg/kg), GSK’872 (10 mg/kg) or vehicle control the day before I/R surgery. **b** Representative images of heart sections from the indicated groups of mice stained with Evans blue and TTC at 24 h after myocardial-ischemia‒reperfusion injury. **c** The ratios of the area at risk (AAR) to the left ventricle (LV) and the infarct area (IA) to the AAR were calculated via Evans blue and TTC staining (*n* = 6). **d** The serum levels of lactate dehydrogenase (LDH, U/L) in the indicated groups of mice 24 h after I/R surgery (*n* = 8). **e** Representative images and quantitative analysis of myocardial Evans blue dye (EBD) uptake and viable cardiomyocytes labeled with α-actinin antibodies in *Myh6*^+^ and *Zbp1*^*fl/fl*^*Myh6*^+^ mice 24 h after I/R surgery (*n* = 10; scale bar = 100 μm). **f** Representative TUNEL staining and relative quantification of heart samples from *Myh6*^+^ and *Zbp1*^*fl/fl*^*Myh6*^+^ mice 24 h after I/R surgery (*n* = 10; scale bar = 20 μm). **g** Western blot and quantification of ZBP1, total caspase 3 (T-Caspase 3), cleaved caspase 3 (Cl-Caspase 3), phosphorylated-RIPK3 (p-RIPK3), RIPK3, full-length GSDMD (GSDMD-FL) and GSDMD-N fragment protein levels in heart samples from *Myh6*^+^ and *Zbp1*^*fl/fl*^*Myh6*^+^ mice 24 h after I/R surgery (*n* = 4). For all the statistical plots, the data are presented as the means ± SEMs. **c**, **d** by one-way ANOVA with Bonferroni multiple comparison test. **e**, **f** by two-tailed unpaired Student’s t-test. **g** by two-way ANOVA with Bonferroni multiple comparison test

To verify the occurrence of PANoptosis-like cardiomyocyte death and the involvement of ZBP1 in I/R injury in vivo, 8-week-old *Zbp1*^*fl/fl*^*Myh6*^+^ and *Myh6*^*+*^ mice were subjected to 30 min of myocardial ischemia followed by 24 h of reperfusion (Fig. 3a). The mice received intraperitoneal injections of the cell death inhibitors GSK’872 and z-VAD-FMK (zVAD) or vehicle control 6 h prior to I/R surgery. GSK’872 was used to inhibit RIPK3-mediated necroptosis, and zVAD was used to inhibit apoptosis as well as CASP1- or CASP11-mediated pyroptosis. I/R-induced myocardial injury was assessed by triphenyltetrazolium chloride (TTC) staining. Although GSK’872 and zVAD decreased the size of the infarct region, ZBP1 deficiency resulted in an even greater decrease (Fig. 3b, c). Additionally, I/R-induced LDH release was most significantly reduced in the *Zbp1*^*fl/fl*^*Myh6*^+^ mice (Fig. 3d). These findings suggest that ZBP1 deficiency substantially improves myocardial I/R injury to a greater extent than individual cell death inhibitors do, indicating that ZBP1 likely functions further upstream as a regulator of pyroptosis, apoptosis, and necroptosis. Evans blue dye (EBD) and TUNEL staining were subsequently used to delineate the specific effects of ZBP1 on I/R-induced cardiomyocyte death. The findings revealed that the *Zbp1*^*fl/fl*^*Myh6*^+^ mice exhibited greater resistance to I/R-induced myocardial necrosis and apoptosis than the *Myh6*^*+*^ mice did (Fig. 3e, f). Additionally, Western blot analysis demonstrated that ZBP1 deficiency significantly inhibited the activation of multiple cell-death proteins, including CASP3, RIPK3, and gasdermin D (GSDMD) (Fig. 3g). These results indicate that the protective effects of ZBP1 deficiency can be attributed to its inhibitory impact on I/R-induced PANoptosis-like cardiomyocyte death.

Next, we evaluated cardiac function and adverse cardiac remodeling in *Zbp1*^*fl/fl*^*Myh6*^+^ and *Myh6*^*+*^ mice 28 days after I/R injury. Echocardiographic analysis revealed that, compared with *Myh6*^*+*^ control mice, *Zbp1*^*fl/fl*^*Myh6*^+^ mice presented a significant overall increase in the left ventricular ejection fraction (LVEF) and an overall reduction in the left ventricular internal diameter (LVID) (Supplementary Fig. 7a–d). Masson’s trichrome staining demonstrated that the *Zbp1*^*fl/fl*^*Myh6*^+^ mice exhibited significantly reduced I/R-induced cardiac fibrosis (Supplementary Fig. 7e). At the molecular level, ZBP1 deficiency significantly decreased the expression of the heart failure marker brain natriuretic peptide (BNP) and the fibrotic marker collagen I in the ischemic regions of *Zbp1*^*fl/fl*^*Myh6*^+^ mice (Supplementary Fig. 7f). These data suggest that *Zbp1* knockout also confers beneficial long-term effects following I/R injury.

Considering the high expression of ZBP1 in immune cells and the substantial inflammatory response triggered by I/R, we generated myeloid-specific ZBP1-deficient mice by crossing *Zbp1*^*fl/fl*^ mice with Lyz2^Cre^ mice (*Zbp1*^*fl/fl*^ × Lyz2^+^ mice) to examine whether myeloid-specific ZBP1 deficiency also influences the pathogenic alterations associated with I/R injury (Supplementary Fig. 8a). qRT‒PCR confirmed the efficient knockout of ZBP1 in bone marrow (BM) cells (Supplementary Fig. 8b). However, BM cell-specific ZBP1 deficiency did not reduce infarct size or LDH release following I/R injury (Supplementary Fig. 8c, d), suggesting that ZBP1 from BM-derived cells barely contributes to the cardiomyocyte death induced by I/R.

### Cardiomyocyte-specific overexpression of *Zbp1* leads to cardiac remodeling and heart failure

**Fig. 4:**
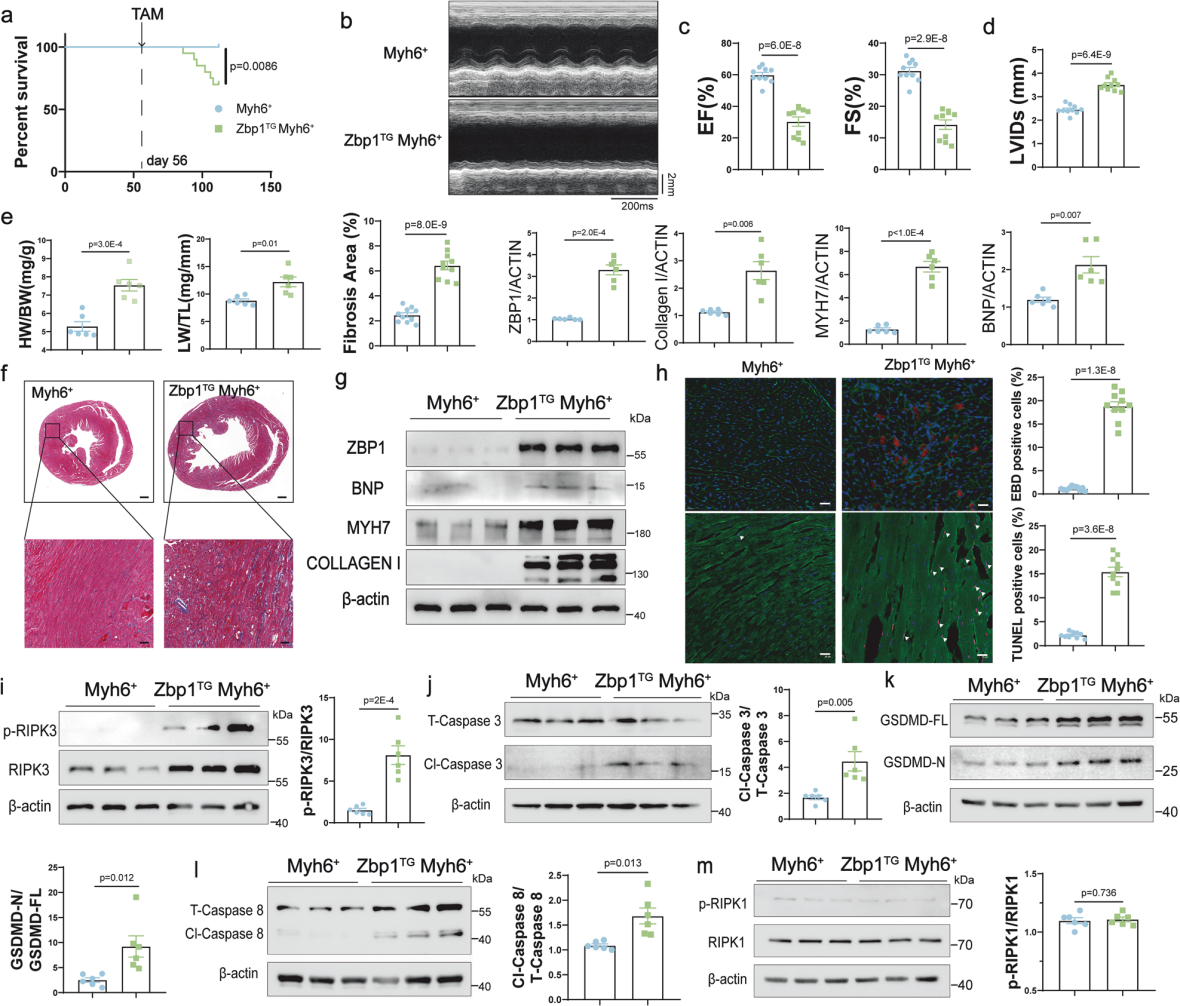
Cardiomyocyte-specific overexpression of *Zbp1* leads to cardiac remodeling and heart failure. **a** The survival rates of *Myh6*^+^ and *Zbp1*^*TG*^*Myh6*^*+*^ mice were estimated via the Kaplan‒Meier method. **b** M-mode echocardiography of *Myh6*^*+*^ and *Zbp1*^*TG*^*Myh6*^*+*^ mice 8 weeks post-tamoxifen injection. **c**, **d** Left ventricular EF, FS (**c**) and LVID_s_ (**d**) were assessed by echocardiography in *Myh6*^*+*^ and *Zbp1*^*TG*^*Myh6*^*+*^ mice 8 weeks after tamoxifen injection (*n* = 10). **e** Ratios of HW to BW and LW to TL in *Myh6*^*+*^ and *Zbp1*^*TG*^*Myh6*^*+*^ mice 8 weeks after tamoxifen injection (*n* = 6). **f** Representative images and quantitative analysis of Masson staining of heart sections from *Myh6*^*+*^ and *Zbp1*^*TG*^*Myh6*^*+*^ mice 8 weeks post-tamoxifen injection (*n* = 10; scale bar = 500 µm in the upper panel and 50 µm in the lower panel). **g** Western blot and quantification of ZBP1, BNP, MYH7 and Collagen I protein levels in heart samples from *Myh6*^+^ and *Zbp1*^*TG*^*Myh6*^*+*^ mice (*n* = 6). **h** Representative photographs and quantitative analysis of myocardial Evans blue dye (EBD) uptake and WGA (wheat germ agglutinin) staining in *Myh6*^*+*^ and *Zbp1*^*TG*^*Myh6*^*+*^ mice 8 weeks post-tamoxifen injection (*n* = 10; scale bar = 20 μm). Representative TUNEL staining and relative quantification of heart samples from *Myh6*^+^ and *Zbp1*^*TG*^*Myh6*^*+*^ mice 8 weeks post-tamoxifen injection (*n* = 10; scale bar = 20 μm). **i** Western blot and quantification of phosphorylated-RIPK3 (p-RIPK3) and RIPK3 protein levels in cardiomyocytes isolated from *Myh6*^+^ and *Zbp1*^*TG*^*Myh6*^*+*^ mice 8 weeks after tamoxifen injection (*n* = 6). **j** Western blot and quantification of total caspase 3 (T-Caspase 3) and cleaved caspase 3 (Cl-Caspase 3) protein levels in cardiomyocytes isolated from *Myh6*^+^ and *Zbp1*^*TG*^*Myh6*^*+*^ mice 8 weeks after tamoxifen injection (*n* = 6). **k** Western blot and quantification of full-length GSDMD (GSDMD-FL) and GSDMD-N fragment protein levels in cardiomyocytes isolated from *Myh6*^+^ and *Zbp1*^*TG*^*Myh6*^*+*^ mice 8 weeks after tamoxifen injection (*n* = 6). **l** Western blot and quantification of total caspase 8 (T-Caspase 8) and cleaved caspase 8 (Cl-Caspase 8) protein levels in cardiomyocytes isolated from *Myh6*^+^ and *Zbp1*^*TG*^*Myh6*^*+*^ mice 8 weeks after tamoxifen injection (*n* = 6). **m** Western blot and quantification of phosphorylated RIPK1 (p-RIPK1) and RIPK1 protein levels in cardiomyocytes isolated from *Myh6*^+^ and *Zbp1*^*TG*^*Myh6*^*+*^ mice 8 weeks post-tamoxifen injection (*n* = 6). For all the statistical plots, the data are presented as the means ± SEMs. **a** by the log-rank test. **c**–**e** and **i**–**m** by two-tailed unpaired Student’s t-test. **f**–**h** by Welch’s t-test. EF ejection fraction, FS fractional shortening, LVID_s_ left ventricular internal diameter at end-systole, HW/BW ratio of heart weight to body weight, LW/TL ratio of lung weight to tibia length

Considering the protective effects of ZBP1 deficiency during myocardial I/R injury, we next evaluated whether any adverse effects occurred in response to ZBP1 overexpression. For this purpose, *Zbp1*-transgenic (*Zbp1*^TG^) mice were crossed with *Myh6*-Cre^ERT2^ mice to generate cardiomyocyte-specific *Zbp1*-overexpressing (*Zbp1*^TG^*Myh6*^+^) mice (Supplementary Fig. 9a). PCR analysis was used to genotype the mice (Supplementary Fig. 9b). Following 5 days of TAM administration (50 mg/kg), ZBP1 mRNA levels were evaluated in *Zbp1*^TG^*Myh6*^+^ mice to confirm successful overexpression (Supplementary Fig. 9c). Cardiomyocyte-specific overexpression was further validated by Western blot and immunofluorescence staining analysis (Supplementary Fig. 9d, e). At 8–10 weeks of age and at baseline, there were no significant differences in cardiac morphology or function between the *Zbp1*^TG^*Myh6*^+^ mice and their *Myh6*^+^ controls. ZBP1 overexpression had no effect on survival until 4 weeks after TAM administration (Fig. 4a). Echocardiography and histological analyses were performed on surviving *Zbp1*^TG^*Myh6*^+^ mice 8 weeks after TAM administration. The echocardiographic assessment revealed a significant overall reduction in LVEF and an increase in LVIDs in the *Zbp1*^TG^*Myh6*^+^ mice compared with the *Myh6*^*+*^ control mice (Fig. 4b–d). Moreover, ZBP1 overexpression led to a significant overall increase in the heart weight-to-body weight (HW/BW) and lung weight-to-tibia length (LW/TL) ratios in these mice (Fig. 4e). Masson’s trichrome staining revealed excessive collagen deposition in the hearts of the *Zbp1*^TG^*Myh6*^+^ mice, indicating increased cardiac fibrosis, a hallmark of pathological cardiac remodeling (Fig. 4f). At the molecular level, cardiomyocyte-specific ZBP1 overexpression significantly upregulated BNP, Myosin-7 (MYH7), and collagen I in left ventricular cardiac tissues (Fig. 4g). Furthermore, EBD and TUNEL staining revealed a significant increase in the number of necrotic and apoptotic cardiomyocytes in the *Zbp1*^TG^*Myh6*^+^ mice (Fig. 4h). Taken together, these data suggest that myocardial *Zbp1* overexpression in adult mice induces cardiac remodeling and heart failure, which are likely driven by cardiomyocyte death.

### Cardiomyocyte-specific overexpression of *Zbp1* directly induces PANoptosis-like cardiomyocyte death in vivo

To explore the mechanisms underlying ZBP1 overexpression-induced cardiac remodeling in vivo, comprehensive RNA-seq analyses were conducted on cardiomyocytes isolated from *Zbp1*^TG^*Myh6*^+^ and *Myh6*^*+*^ mice 8 weeks after TAM administration. PCA revealed a distinct separation of gene expression profiles between the CMs from *Zbp1*^TG^*Myh6*^+^ and *Myh6*^*+*^ mice (Supplementary Fig. 10a). Differentially expressed gene (DEG) analysis revealed 2646 and 2345 significantly up- and downregulated genes (false discovery rate (q value) <0.05; fold change >1.5 or fold change <1/1.5) in the samples from *Zbp1*^TG^*Myh6*^+^ mice compared with those from *Myh6*^+^ mice (Supplementary Fig. 10b). GO analysis indicated that the upregulated genes were enriched primarily in necrotic cell death, neuron death and the apoptotic signaling pathway (Supplementary Fig. 10c). In contrast, the downregulated genes were notably associated with muscle cell differentiation and fatty acid metabolism (Supplementary Fig. 10d). Notably, the IFN-I and NFκB signaling pathways were surprisingly not significantly enriched, which may reflect a distinct feature of adult cardiomyocytes. These findings collectively suggest that *Zbp1* overexpression mainly induces cardiomyocyte death instead of inflammation. To verify these results, Western blot analysis was performed, which revealed that *Zbp1* overexpression significantly promoted the activation of RIPK3, CASP3, and GSDMD (Fig. 4i–k), indicating the sufficiency of *Zbp1* overexpression to drive cardiomyocyte PANoptosis. Next, we explored whether ZBP1 regulated other proteins involved in PANoptosis, including CASP8 and RIPK1. Notably, ZBP1 overexpression elevated the protein levels of both full-length and cleaved CASP8 (Fig. 4l), suggesting a potential critical role of CASP8 in ZBP1-mediated PANoptosis. However, ZBP1 overexpression had no effect on the expression of phosphorylated RIPK1 (Fig. 4m). Consistently, the I/R-induced upregulation of cleaved CASP8 was significantly inhibited by ZBP1 deficiency (Supplementary Fig. 11a), and no decrease in phosphorylated RIPK1 was observed (Supplementary Fig. 11b). Collectively, these findings suggest that *Zbp1* overexpression contributes to cardiac dysfunction by driving PANoptosis-like cell death in cardiomyocytes.

### ZBP1 induces cardiomyocyte PANoptosis by modulating noncanonical PANoptosome assembly

Next, we investigated whether *Zbp1* overexpression was sufficient to induce PANoptosis-like cardiomyocyte death in vitro. Adult cardiomyocytes from WT mice were infected with control adenovirus (Ad-NC) or *Zbp1*-overexpressing adenovirus (Ad-*Zbp1*). ZBP1 overexpression significantly increased the number of TUNEL-positive and PI-positive cardiomyocytes as well as the presence of balloon-shaped vesicles (yellow arrow) (Supplementary Fig. 12a, b). These findings indicate that ZBP1 can directly induce both apoptotic and nonapoptotic forms of cell death in cardiomyocytes. Next, a series of inhibitors targeting apoptosis, pyroptosis, and necroptosis were used to investigate the specific pathways involved in ZBP1 overexpression-induced cardiomyocyte death. zVAD significantly inhibited ZBP1 overexpression-induced apoptosis, as evidenced by the increase in CASP3 activity (Supplementary Fig. 12c). Although individual LDC7559, GSK’872 or zVAD treatment reduced the LDH release caused by ZBP1 overexpression, the combination of zVAD with GSK’872 had a better protective effect (Supplementary Fig. 12d). Furthermore, ZBP1 overexpression-induced the release of the inflammatory factor IL-18, which is associated with pyroptosis, was significantly inhibited by LDC7559, GSK’872 or zVAD treatment (Supplementary Fig. 12e). Notably, Nec-1 had no significant effect on ZBP1-induced cell death, indicating that RIPK1 may not be required for this process.

**Fig. 5:**
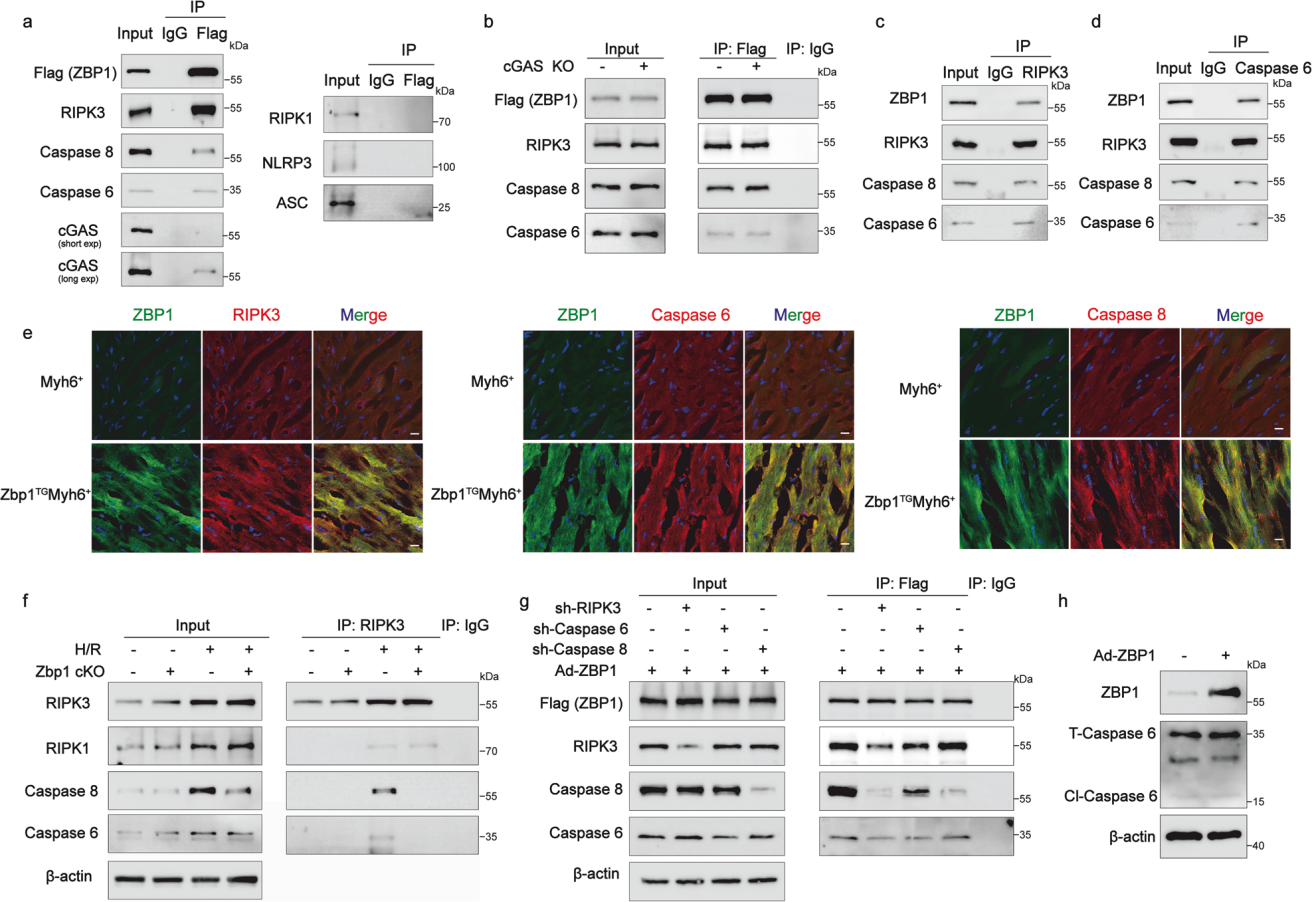
ZBP1 Induces cardiomyocyte PANoptosis by modulating noncanonical PANoptosome assembly. **a** Coimmunoprecipitation and Western blot assays of Flag (ZBP1) and RIPK3, Caspase 8, Caspase 6, cGAS, RIPK1, NLRP3, and ASC interaction in cardiomyocytes infected with adenovirus expressing ZBP1. **b** Coimmunoprecipitation and Western blot assays of Flag (ZBP1) and RIPK3, Caspase 8, and Caspase 6 in cardiomyocytes isolated from WT or *cGAS*^-/-^ mice infected with adenovirus expressing ZBP1. **c** Coimmunoprecipitation and Western blot assays of RIPK3 and ZBP1, Caspase 8, and Caspase 6 interaction in cardiomyocytes infected with adenovirus expressing ZBP1. **d** Coimmunoprecipitation and Western blot assays of the interaction between Caspase 6 and ZBP1, RIPK3, and Caspase 8 in cardiomyocytes infected with adenovirus expressing ZBP1. **e** Representative confocal images of ZBP1 and RIPK3, Caspase 8, Caspase 6 colocalization in heart sections from *Myh6*^+^ and *Zbp1*^*TG*^*Myh6*^*+*^ mice 8 weeks after tamoxifen injection (scale bar = 10 μm). **f** Coimmunoprecipitation and Western blot analyses of RIPK3 and RIPK1, Caspase 8, and Caspase 6 interactions in adult *Myh6*^+^ and *Zbp1*^*fl/fl*^*Myh6*^+^ mouse cardiomyocytes subjected to H/R for 0.5/24 h. **g** Coimmunoprecipitation and Western blot assays of Flag (ZBP1) and RIPK3, Caspase 6, and Caspase 8 interaction in cardiomyocytes infected with adenovirus expressing sh-RIPK3, sh-Caspase 6 or sh-Caspase 8 with ZBP1 overexpression. **h** Western blot of ZBP1, total caspase 6 (T-Caspase 6) and cleaved caspase 6 (Cl-Caspase 6) in cardiomyocytes infected with adenovirus expressing ZBP1 or the NC

Having confirmed the existence of ZBP1-mediated cardiomyocyte PANoptosis, we next aimed to identify the PANoptosome complex that can simultaneously regulate the three cell-death pathways. Given that previous studies have identified key components of the ZBP1 PANoptosome in infectious diseases,^22^ we aimed to confirm whether these molecules may also participate in PANoptosome assembly in cardiomyocytes. Immunoprecipitation analysis demonstrated that ZBP1 overexpression in cardiomyocytes facilitated its interaction with RIPK3, CASP8, and CASP6. However, no interactions were observed with RIPK1, NLR family pyrin domain-containing protein 3 (NLRP3), or the apoptosis-associated speck-like protein CARD (ASC) (Fig. 5a), indicating that ZBP1 overexpression did not activate the NLRP3 inflammasome in cardiomyocytes, unlike its effects in immune cells. Interestingly, although a modest interaction between ZBP1 and cGAS was observed (Fig. 5a), the deletion of cGAS did not affect the interactions among ZBP1, RIPK3, CASP8, and CASP6 (Fig. 5b), suggesting that cGAS is not essential for the formation of the ZBP1/RIPK3/CASP8/CASP6 complex. Coimmunoprecipitation assays using the RIPK3 and CASP6 proteins as bait further confirmed the physical interactions among ZBP1, RIPK3, CASP8, and CASP6 (Fig. 5c, d). Immunofluorescence staining also revealed the colocalization of ZBP1 with RIPK3, CASP8, and CASP6 in *Zbp1*^TG^*Myh6*^+^ mice 8 weeks after TAM administration (Fig. 5e). To further confirm the critical role of ZBP1 in PANoptosome assembly under H/R stress, we conducted an immunoprecipitation assay in which the RIPK3 protein was used as bait in cardiomyocytes isolated from *Zbp1*^*fl/fl*^*Myh6*^+^ and *Myh6*^*+*^ mice subjected to 24 h of H/R. The results revealed interactions of RIPK3 with CASP8 and CASP6 following H/R, whereas these interactions were absent in *Zbp1*^*fl/fl*^*Myh6*^+^ cardiomyocytes (Fig. 5f). However, the interactions between RIPK3 and RIPK1 remained unaffected by ZBP1 deficiency, which was consistent with our previous findings indicating that the activation of RIPK1 occurred independently of ZBP1. Furthermore, knockdown of RIPK3 and CASP6 disrupted the interaction between ZBP1 and other PANoptosome components, suggesting their critical role in complex formation (Fig. 5g). In contrast, CASP8 knockdown selectively impaired its own incorporation into the PANoptosome without markedly affecting other interactions (Fig. 5g). Notably, ZBP1 overexpression did not alter CASP6 cleavage, suggesting that the cleavage of CASP6 may not be required for ZBP1-mediated PANoptosome assembly (Fig. 5h). Collectively, these findings indicate that ZBP1 drives cardiomyocyte death by promoting the formation of the ZBP1/RIPK3/CAS8/CAS6 PANoptosome complex and that the protective effects of ZBP1 deficiency may be partially attributed to the inhibition of PANoptosome assembly.

Previous studies have demonstrated that RIPK3 can mediate cardiomyocyte necroptosis through the activation of CaMKII in myocardial I/R injury.^23,24^ Accordingly, we examined whether CaMKII activation is also regulated by ZBP1. Coimmunoprecipitation assays revealed that ZBP1 overexpression markedly enhanced the physical interaction between RIPK3 and CaMKII in cardiomyocytes (Supplementary Fig. 13a). Furthermore, ZBP1 overexpression significantly increased the level of phosphorylated CaMKII (Supplementary Fig. 13b), whereas ZBP1 deficiency attenuated the I/R-induced increase in phosphorylated CaMKII (Supplementary Fig. 13c). Collectively, these findings suggest that ZBP1-induced cardiomyocyte death is mediated, at least in part, through CaMKII activation.

### Cell death inhibitors ameliorate the cardiac remodeling induced by *Zbp1* overexpression

To determine whether cardiomyocyte death is responsible for the cardiac remodeling induced by *Zbp1* overexpression, *Zbp1*^TG^*Myh6*^+^ and *Myh6*^*+*^ mice were treated with Nec-1, GSK’872, zVAD, or vehicle control. Because the *Zbp1*^TG^*Myh6*^+^ mice did not exhibit any significant changes in cardiac function 4 weeks after TAM administration, we administered these inhibitors intraperitoneally every day for 28 days, from 4 to 8 weeks after TAM administration. Echocardiography and histological analyses were performed for each group of mice 8 weeks after TAM administration. Echocardiographic analysis revealed that although individual GSK’872 or zVAD treatment ameliorated cardiac dysfunction and left ventricular chamber dilatation in *Zbp1*^TG^*Myh6*^+^ mice, combined treatment with both inhibitors resulted in more pronounced attenuation (Supplementary Fig. 14a–c). Consistently, Masson’s trichrome staining revealed significantly less collagen deposition in the group of *Zbp1*^TG^*Myh6*^+^ mice treated with GSK’872 plus zVAD than in the other treatment groups (Supplementary Fig. 14d). Furthermore, EBD staining revealed that combined treatment with GSK’872 and zVAD resulted in the most significant reduction in the number of EBD-positive cardiomyocytes (Supplementary Fig. 14e). As expected, Nec-1 had no significant effect on the cardiac dysfunction or remodeling induced by *Zbp1* overexpression (Supplementary Fig. 14a–e), which was consistent with our previous findings in vitro. However, *Zbp1* overexpression resulted in the surprising upregulation of ISGs in heart tissues, which was attenuated by all the cell death inhibitors (Supplementary Fig. 14f). These findings suggest that the inflammatory response may be secondary to cardiomyocyte death rather than driven by cardiomyocyte inflammation itself. Collectively, these results indicate the parallel contributions of pyroptosis, apoptosis, and necroptosis to ZBP1-induced cardiac remodeling.

### MSB is a novel ZBP1 inhibitor that protects against myocardial ischemia‒reperfusion injury

**Fig. 6:**
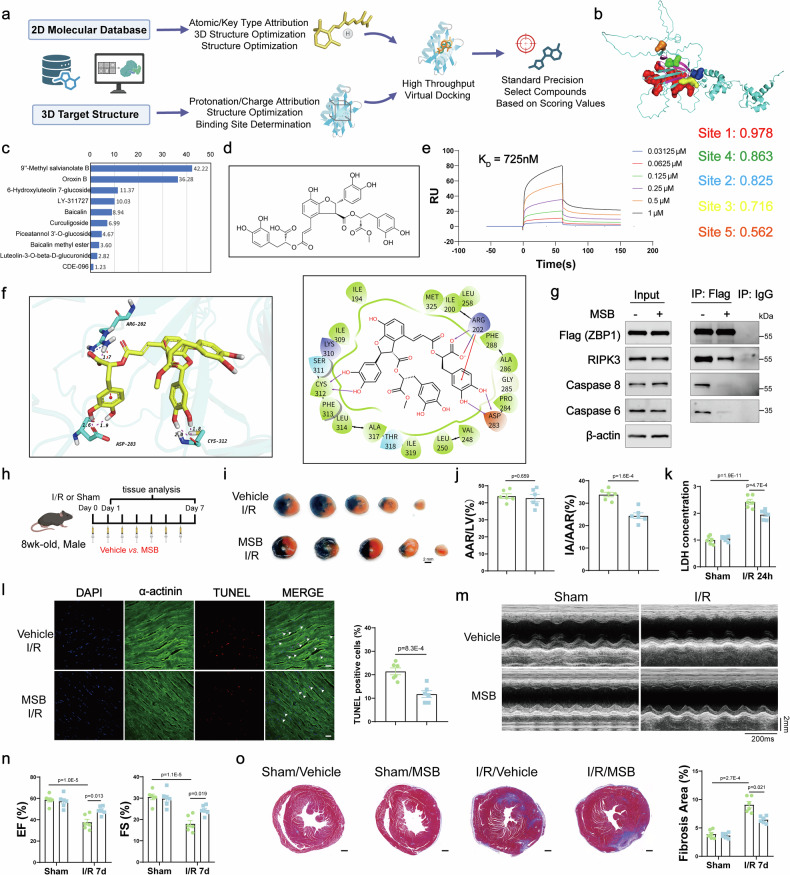
MSB is a novel ZBP1 inhibitor that protects against myocardial ischemia‒reperfusion injury. **a** Schematic diagram displaying a strategy to screen mouse ZBP1 inhibitors. **b** The druggable binding pockets of mouse ZBP1 (AlphaFold ID: AF-Q9QY24-F1) were predicted and scored on the basis of its 3D structure. Virtual screening was performed according to site 4. **c** Surface plasmon resonance (SPR) analysis of the binding response of ten compounds with ZBP1 at a single concentration (50 µM). **d** The chemical structure of MSB. **e** Kinetic curves from subsequent concentration-dependent SPR analysis showing the binding of MSB to ZBP1. **f** 2D and 3D patterns of MSB and mouse ZBP1 binding. **g** Coimmunoprecipitation and Western blot assays of Flag (ZBP1) and RIPK3, Caspase 6, and Caspase 8 interactions in cardiomyocytes infected with adenovirus expressing ZBP1 with or without MSB (1 μM) treatment. **h** Schematic representation of the experimental protocol for myocardial I/R injury in mice treated with vehicle or MSB (5 mg/kg/day, 2 h before the onset of ischemia for the first time). **i** Representative images of heart sections stained with Evans blue and TTC from WT mice 24 h after I/R surgery and treated with MSB or vehicle. **j** The ratios of the area at risk (AAR) to the left ventricle (LV) and the infarct area (IA) to the AAR were calculated via Evans blue and TTC staining (*n* = 6). **k** Serum levels of lactate dehydrogenase (LDH, U/L) in WT mice 24 h after I/R surgery treated with MSB or vehicle (*n* = 6). **l** Representative TUNEL staining and relative quantification of heart samples from WT mice 24 h after I/R surgery and treated with MSB or vehicle (*n* = 6; scale bar = 20 μm). **m** M-mode echocardiography of WT mice 7 days post-I/R injury treated with MSB or vehicle. **n** Left ventricular EF and FS were assessed by echocardiography in WT mice 7 days post-I/R injury treated with MSB or vehicle (*n* = 6). **o** Representative images and quantitative analysis of Masson staining of heart sections from WT mice 7 days post-I/R injury treated with MSB or vehicle (*n* = 6, scale bar = 500 μm). For all the statistical plots, the data are presented as the means ± SEMs. **j** and **l** by two-tailed unpaired Student’s t-test. **k** and **n**–**o** by two-way ANOVA with Bonferroni multiple comparison test. EF ejection fraction, FS fractional shortening

Given the potential of ZBP1 as a therapeutic target for myocardial I/R injury, we performed structure-based virtual screening to identify novel small-molecule inhibitors of ZBP1, with the specific aim of disrupting RHIM-RHIM domain-mediated interactions with partner proteins (Fig. 6a). As the high-resolution crystal structure of the ZBP1 RHIM domain has not yet been resolved, we utilized the mouse ZBP1 structure predicted by AlphaFold, which presented higher confidence scores and superior structural reliability than the human homolog. Using Schrödinger software, five druggable binding pockets were predicted, among which site 4 was selected for docking because it covered the largest number of residues within the RHIM domain (Fig. 6b). A total of 300,000 compounds from the MedChemExpress (MCE) library were docked against the ZBP1 structure, and the top ten compounds were selected for further evaluation on the basis of docking scores. Surface plasmon resonance (SPR) screening at a single concentration (50 µM) revealed that MSB exhibited the strongest binding response to the immobilized ZBP1 protein (response unit (RU) = 42.2) (Fig. 6c, d), suggesting its potential as a lead ZBP1-binding molecule. Subsequent concentration-dependent SPR analysis confirmed the interaction, yielding a dissociation constant (K_D_) of 725 nM, indicative of moderate-to-high binding affinity (Fig. 6e, f). Coimmunoprecipitation assays revealed that the interactions of ZBP1 with RIPK3, CASP8, and CASP6 were attenuated following MSB treatment in cardiomyocytes (Fig. 6g). These findings suggest that MSB effectively disrupts ZBP1-mediated PANoptosome assembly.

To further elucidate the cardioprotective effect of MSB in the context of I/R injury, adult cardiomyocytes were isolated and subjected to H/R stress. MSB treatment mitigated H/R-induced cardiomyocyte death, as indicated by a reduced number of PI-positive cells, decreased LDH release, and decreased CASP3 activity (Supplementary Fig. 15a–c). In vivo, MSB treatment significantly reduced the I/R-induced increase in myocardial infarct size (Fig. 6h–j). Furthermore, MSB ameliorated cardiomyocyte death, as evidenced by lower serum LDH concentrations and fewer TUNEL-positive cells in cardiac tissue (Fig. 6k, l). The functional relevance of MSB was further assessed at 7 days post-I/R injury. Echocardiographic analysis revealed that MSB attenuated the decrease in LVEF (Fig. 6m, n), whereas Masson’s trichrome staining demonstrated a reduction in myocardial fibrosis (Fig. 6o). These findings collectively suggest the therapeutic potential of MSB in mitigating myocardial injury and adverse remodeling following I/R insult.

## Discussion

To the best of our knowledge, this study constitutes the initial documentation of ZBP1-induced PANoptosis in adult cardiomyocytes. Here, by conducting a comprehensive bioinformatics analysis, we identified an essential role of ZBP1 in myocardial I/R injury, particularly during the reperfusion phase. Additionally, we found that H/R stress induces cardiomyocyte pyroptosis, apoptosis, and necroptosis, all of which contribute to myocardial I/R injury and can be mitigated by *Zbp1* deficiency in mice. Furthermore, this study is the first to investigate the impact of cardiomyocyte-specific *Zbp1* overexpression in vivo, allowing for a more specific evaluation of the role of ZBP1 in inducing cardiomyocyte PANoptosis. More importantly, virtual screening and experimental validation revealed a novel small-molecule compound, MSB, which has high binding affinity for ZBP1 and effectively attenuates myocardial I/R injury both in vitro and in vivo. Collectively, these findings demonstrate that ZBP1 is a novel mediator of cardiomyocyte PANoptosis that may serve as a new therapeutic target for myocardial I/R injury.

ZBP1 was initially identified as an innate immune sensor that activates the NF-κB signaling pathway and the IFN-I response.^14^ Previously reported data have provided some insights into the role of ZBP1 in cardiac inflammation; however, they have yielded distinct and sometimes contradictory results. Enzan et al. revealed that global knockout of ZBP1 exacerbates mtDNA-induced inflammation by activating the NF-κB signaling pathway following MI,^25^ whereas Lei et al. demonstrated that ZBP1 senses mtDNA and contributes to doxorubicin-induced cardiotoxicity by sustaining cardiac interferon type I (IFN-I) responses.^26^ However, both studies utilized systemic *Zbp1* knockout mice and isolated neonatal cardiomyocytes, leaving a gap in understanding the specific role of ZBP1 in adult cardiomyocytes. Furthermore, Enzan et al. reported that ZBP1 expression was elevated in noninfarcted regions of hearts following nonreperfused myocardial infarction. In contrast, our findings demonstrate that ZBP1 is predominantly upregulated in infarcted regions during myocardial ischemia–reperfusion (I/R) injury, particularly during the reperfusion phase. This discrepancy is likely attributed to the pathophysiological distinction between reperfused and nonreperfused myocardial infarction.^27^ Notably, previous studies have shown that necroptotic signaling mediated by RIPK3 activation is confined to infarcted regions, whereas noninfarcted areas exhibit RIPK3 activation associated with inflammatory responses but without the execution of necroptosis.^28^ On the basis of this evidence, we propose that ZBP1 may mediate distinct cellular responses in infarcted versus noninfarcted myocardial tissue. These results highlight the complex role of ZBP1 in response to various detrimental stimuli and in different cell types. Further investigations are needed to explore the crosstalk between different cell types in the heart to provide a comprehensive understanding of ZBP1 function.

It is widely accepted that ZBP1 activation is triggered by nucleic acid ligands, such as viral nucleic acids from pathogens^20,29^ and damaged endogenous mitochondrial DNA.^26,30^ Other nucleic acid sensors, including those in the cGAS-STING pathway, have also been reported to regulate ZBP1 activation.^26,31^ However, our findings demonstrated that direct overexpression of ZBP1, both in vitro and in vivo, was sufficient to induce cardiomyocyte PANoptosis without the involvement of any nucleic acid ligands. These findings are consistent with previous studies showing that ZBP1 can function as an adapter protein for AIM2 or the TLR3/4-TRIF complex to facilitate PANoptosis signaling even without ligand binding.^6,32^ Another study reported that ZBP1, without binding to nucleic acid ligands, can also trigger cell death under heatstroke conditions,^33^ further supporting our observations. Furthermore, we did not observe regulatory interactions between ZBP1 and the cGAS-STING pathway, suggesting potential differences in the role of ZBP1 depending on the presence or absence of nucleic acid ligands.

Previous evidence indicates that the activation of PANoptosis is regulated by a single cell death complex known as the PANoptosome, which coordinates the interactions of key components from each cell death pathway.^22^ Although the specific composition of this complex may vary depending on the stimulus, it generally includes sensors, adapters, and effectors.^22^ ZBP1 serves as a representative sensor that can interact with various proteins through its Zα and RHIM domains. The RHIM domains enable ZBP1 to interact with RIPK1 and RIPK3.^11^ The ZBP1/RIPK1 complex reportedly transduces NF-κB signaling during microbial infections,^19^ whereas the ZBP1/RIPK3 complex recruits other components necessary for PANoptosome assembly.^20,34^ Here, we demonstrated that ZBP1, RIPK3, CASP8, and CASP6 interact to form a PANoptosome and that some canonical components, such as RIPK1, NLRP3, and ASC, are dispensable for the formation of this ZBP1-derived PANoptosome in cardiomyocytes. These findings can be attributed to the distinct expression patterns of canonical inflammasome components in cardiomyocytes compared with those in other cell types, resulting in a simpler PANoptosome in cardiomyocytes than in immune cells. Our results are consistent with those of previous studies from our group and others, which suggest that NLRP3 inflammasome activation and interleukin-1 beta (IL-1β) release primarily occur in non-cardiomyocytes,^35,36^ whereas cardiomyocytes undergo CASP11/GSDMD-dependent, noncanonical pyroptosis in response to myocardial I/R injury.^37^ The requirement of CASP6, rather than RIPK1, for the formation of the ZBP1-derived PANoptosome offers novel insights into the intricate relationships among CASP6, RIPK1, RIPK3, and ZBP1. It has been proposed that CASP6 enhances the interaction between ZBP1 and RIPK3 by binding to RIPK3 during IAV infection.^34^ In contrast, RIPK1 inhibits the formation of the ZBP1/RIPK3 complex, thereby preventing necroptosis and skin inflammation.^38,39^ It is possible that the formation of the ZBP1/RIPK3/CASP6 complex enhances its effectiveness in inducing cardiomyocyte PANoptosis. However, the interactions among RIPK3, CASP6, and CASP8 are inhibited in the absence of ZBP1, indicating that ZBP1 is a central component of the PANoptosome. Although RIPK1 appears to play a minimal role in the cardiac dysfunction and cardiomyocyte death induced by *Zbp1* overexpression, its inhibition with Nec-1 suppresses IFN-I responses, indicating a potential role in non-cardiomyocytes that warrants further investigation.

Cardiomyocyte death is a critical factor in the pathogenesis of myocardial I/R injury. However, targeting a single type of cell death has been reported to be ineffective at preventing I/R injury,^40–42^ and such approaches may even activate alternative cell death pathways.^43,44^ In contrast, the combined inhibition of necroptosis and ferroptosis has been shown to significantly enhance cardioprotection against myocardial I/R injury.^41^ Furthermore, the combined use of the cell death inhibitors necrostatin-1 and zVAD has been demonstrated to result in a more significant reduction in myocardial infarct size in response to I/R than the use of either inhibitor alone.^42^ These results underscore the need to elucidate the extensive crosstalk among cell death pathways and to develop therapies that target multiple forms of cardiomyocyte death to protect against I/R injury. Our study revealed that, compared with conventional cell death inhibitors, such as GSK’872 and zVAD, ZBP1 deficiency reversed the I/R-induced upregulation of PANoptosis-associated proteins, resulting in a more substantial reduction in myocardial infarct size. The development of effective agents that target upstream ZBP1 or the subsequent assembly of the PANoptosome represents a promising strategy for inhibiting cardiomyocyte death. Moreover, previous studies have implicated ZBP1 as a critical mediator of Diquat-induced ferroptosis of endothelial cells through the activation of RIPK3 and subsequent phosphorylation of FSP1.^30^ These findings suggest a potential link between the canonical ZBP1/RIPK3/MLKL signaling axis and an alternative pathway involving FSP1. Given the emerging evidence supporting the role of ferroptosis in myocardial I/R injury, further investigation is warranted to determine whether ZBP1 contributes to ferroptosis in this context and whether this effect is dependent on RIPK3.

**Fig. 7:**
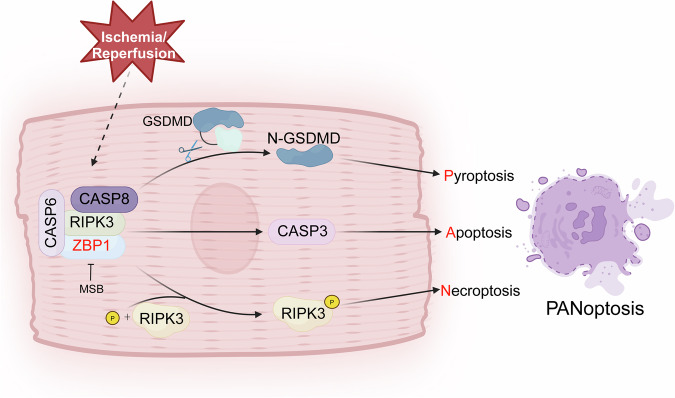
A schematic illustration showing that ZBP1 drives cardiomyocyte PANoptosis through the formation of the ZBP1/RIPK3/CASP6/CASP8 complex. Myocardial I/R injury induced the upregulation of ZBP1 in adult cardiomyocytes. ZBP1 facilitates the assembly of the ZBP1/RIPK3/CAS8/CAS6 PANoptosome complex, thereby driving multiple forms of programmed cell death, including pyroptosis, apoptosis, and necroptosis. MSB exhibits high binding affinity for ZBP1 and effectively attenuates myocardial I/R injury. The schematic illustration was generated using BioRender (https://BioRender.com)

In conclusion, our study revealed that ZBP1 drives cardiomyocyte PANoptosis through the formation of the ZBP1/RIPK3/CASP6/CASP8 complex (Fig. 7). Cardiomyocyte-specific *Zbp1* knockout effectively protects against myocardial I/R injury in mice. These findings may provide new mechanistic insights that may help identify potential therapeutic targets for the treatment of myocardial I/R injury.

## Materials and methods

### Human samples

Ischemic heart tissue samples from patients diagnosed with ischemic cardiomyopathy (ICM) (*n* = 8) were procured during cardiac transplantation procedures. Healthy heart specimens (*n* = 6) were obtained from brain-dead donors with normal circulatory function, who were ineligible for transplantation due to technical or non-cardiac reasons, in accordance with the guidelines established by China Transplant Services. The experimental protocols adhered to the principles outlined in the WMA Declaration of Helsinki and were approved by the ethical review committee of Zhongshan Hospital, Fudan University (B2022-267R). Prior to heart tissue collection, all participants provided written informed consent. Detailed information of ICM patients and healthy controls is provided in Supplementary Table 1.

### Animals

Male C57BL/6 J mice aged 6–8 weeks were purchased from SLAC Laboratory Animal Co. LTD (Shanghai, China). The mice were housed under standard laboratory conditions with a constant temperature of 22 °C, a 12:12-h light: dark cycle, and ad libitum access to tap water and standard laboratory mouse chow. The mice were euthanized by cervical dislocation. Ethical approval for all animal experiments was obtained from the Animal Care and Use Committee at Zhongshan Hospital, Fudan University (SYXK-2021-0022). The experimental procedures were in accordance with the Guidelines for Care and Use of Laboratory Animals published by the US National Institutes of Health (NIH Publication, 8th Edition, 2011).

### Mouse myocardial ischemia-reperfusion model

Mice were induced into anesthesia using 2% isoflurane with 100% oxygen ventilation at a flow rate of 2 L/min. Following a left thoracic incision, the heart was exposed, and a slipknot was carefully placed around the left anterior descending coronary artery (LAD) using a 6-0 silk suture. Confirmation of myocardial ischemia was established by the presence of ST segment elevations on the electrocardiogram. After a 30-min period of ischemia, reperfusion was initiated by releasing the slipknot. Sham-operated mice underwent left thoracotomy without ligation of the LAD. The mice were anaesthetized with sodium pentobarbital (50 mg/kg, i.p.) and heart tissue samples were harvested from the ischemic region of the left ventricular myocardium for subsequent analysis via quantitative real-time polymerase chain reaction (qRT-PCR) and Western blotting.

### Determination of mouse myocardial infarcted size

The assessment of infarct area (IA) and area at risk (AAR) was conducted using 2,3,5-triphenyltetrazolium chloride/Evans Blue (TTC/EB) staining. Following a 24-h reperfusion period, mice were anesthetized and their chests were opened. The coronary artery was re-occluded at the original site of occlusion, and 1% Evans blue dye was injected into the aorta to delineate normal and ischemic areas. Subsequently, the hearts were rinsed in phosphate-buffered saline (PBS) and frozen at −80 °C for 15 min before being sectioned into five 1.2-mm-thick transverse slices. These slices were then incubated in 1% TTC at 37 °C for 15 min to visualize the unstained infarcted regions. The infarct area (pale), AAR (red), and total left ventricular (LV) area of each section (blue) were quantified using Image-Pro Plus 6.0 (NIH, Bethesda, MD) by an observer blinded to the experimental conditions.

### Echocardiography analysis

Transthoracic echocardiography was performed utilizing the VeVo 2100 Imaging System (VisualSonics, Toronto, Canada) to assess cardiac structure and function. Mice were anesthetized with 1–2% isoflurane and positioned in a supine orientation on the echo pad. The heart rates of the mice were monitored and maintained at approximately 500 beats per minute. Two-dimensional (2D) long-axis M-mode measurements were acquired at the level of the papillary muscles. The averaged interventricular septum (IVS) diastolic and systolic thickness (IVSd, IVSs), left ventricular (LV) diastolic and systolic posterior wall thickness (LVPWd, LVPWs), LV diastolic and systolic internal dimensions (LVIDd, LVIDs) were quantified. LV fractional shortening (FS) [(LVIDd – LVIDs)/LVIDd] and LV ejection fraction (EF) [(LV Vol;d−LV Vol;s)/LV Vol;d × 100%] were calculated from these M-mode measurements. Subsequent analysis was conducted by an investigator who was blinded to the treatment allocation and genotype of the mice.

### Adult mouse cardiomyocytes and non-cardiomyocytes isolation

Primary Adult cardiomyocytes (CMs) were isolated from the ventricles of male wild-type and gene-edited mice following a previously described protocol with some modifications.^45^ Briefly, mice were anesthetized, and the heart was exposed before the descending aorta was severed. A total of 7 ml of EDTA buffer (130 mM NaCl, 0.5 mM Na_2_HPO_4_, 5 mM KCl, 10 mM Taurine, 10 mM HEPES, 10 mM D-glucose, 10 mmol/l BDM, 5 mM EDTA, pH 7.8) was steadily injected into the right ventricle. Subsequently, the heart was excised and perfused with 10 ml of EDTA buffer, 3 ml of perfusion buffer (130 mM NaCl, 5 mM KCl, 0.5 mM Na_2_HPO_4_, 10 mM HEPES, 10 mM Taurine, 10 mM D-glucose, 10 mmol/l BDM, 1 mM MgCl_2_, pH 7.8) and 30 ml of pre-warmed collagenase buffer (perfusion buffer with 0.5 mg/ml collagenase II, 0.5 mg/ml collagenase IV and 0.05 mg/ml protease XIV) through the left ventricle. All buffers were filtered through a 0.22-μm filter prior to usage. The cardiac ventricles were then dissociated into pieces using the collagenase buffer and digestion was halted by adding 5 ml of stop buffer (perfusion buffer with 5% FBS). The resulting cell suspension was filtered through a 100-μm filter into a 50 ml tube, where it settled into a pellet through gravity. After removing the supernatant, the pellet with CMs underwent 3 steps of gradient calcium reintroduction and were plated on laminin-coated culture dishes. The media was replaced with M199 culture media (Gibco, 12340-030) after 1 h of plating. All the cells were incubated at 37 °C with 5% CO_2_. The remaining cellular components was collected and then filtrated through 70 μm strainers to obtain non-cardiomyocyte fraction. The supernatant was incubated with CD45 MicroBeads (Cat. No. 130-052-301, Miltenyi Biotec) at a ratio of 10 μL MicroBeads per 1 × 10⁷ total cells for 15 min at 4 °C, followed by magnetic separation using an LD Column (Cat. No. 130-042-901, Miltenyi Biotec) to isolate CD45⁺ immune cells. The CD45^-^ fraction was then incubated with CD31 MicroBeads (Cat. No. 130-097-418, Miltenyi Biotec) under the same conditions and processed through another LD Column to separate CD31⁺ endothelial cells (CD45⁻/CD31⁺). The unlabeled flow-through cells (CD45⁻/CD31⁻) were collected as cardiac fibroblasts (CFs).

### Statistical analysis

Data are presented as mean ± standard error of the mean (SEM) for statistical analysis. Shapiro-Wilk test was applied to assess normality of the distribution of data. Before t-test and analysis of variance (ANOVA), homogeneity was tested among variances by F test (for 2 groups) and Brown-Forsythe test (for >2 groups). For data of 2 groups with normal distribution and equal variances, statistical differences were analyzed using two-tailed Student’s t-test. For data of >2 groups with normal distribution and equal variances, statistical differences were analyzed using one-way ANOVA (one variable involved) and two-way ANOVA (two variables involved) with Bonferroni multiple comparison test. Statistical differences of data with unequal variance were evaluated by Welch’s t-test (for 2 groups) or Welch’s ANOVA with Dunnett’s T3 multiple comparison test (for >2 groups). All statistical analyses were performed with Prism software (GraphPad prism for windows, version 9.0, Nashville, USA). *P* values less than 0.05 was considered as statistically significant.

## Supplementary information

Supplementary methods and figures

## Data Availability

The RNA-sequencing data generated in this study are publicly available in Gene Expression Omnibus (GEO) dataset GSE307385.
