# Supplementary material for: Z-DNA-binding protein 1 exacerbates myocardial ischemia‒reperfusion injury by inducing noncanonical cardiomyocyte PANoptosis
Source: Signal Transduct Target Ther. 2025 Oct 7;10:333. doi: 10.1038/s41392-025-02430-5 (PMC12501347; doi:10.1038/s41392-025-02430-5)
Supplement: Supplementary file 1 — Supplementary methods and figures [file 41392_2025_2430_MOESM1_ESM.docx]

Supplementary Materials for

Z-DNA-binding protein 1 exacerbates myocardial ischemia‒reperfusion injury by inducing noncanonical cardiomyocyte PANoptosis

Xiaokai Zhang^#^, Shuai Song^#^, Zihang Huang^#^, Linqi Zeng^#^, Yining Song, Mohan Li, Chenyan Liu, Fengze Cai, Tongyao Wang, Peng Yu, Junbo Ge*, Aijun Sun*

^#^ These authors contributed equally to this work.

Correspondence to: Junbo Ge, email: [jbge@zs-hospital.sh.cn](mailto:jbge@zs-hospital.sh.cn) or Aijun Sun, email: [sun.aijun@zs-hospital.sh.cn](mailto:sun.aijun@zs-hospital.sh.cn)

**This PDF file includes:**

Materials and Methods

Figures. S1 to S15

Tables S1 to S6

**Materials and Methods**

**Genetically Engineered Mice**

*Zbp1* conditional knockout mouse model was created by GemPharmatech Co., Ltd. (Nanjing, Jiangsu, China) using CRISPR/Cas-mediated genome engineering. The *Zbp1* gene is located on mouse chromosome 2 and has 3 transcripts. According to the structure of Zbp1 gene, exon2-exon8 of *Zbp1-201* (ENSMUST00000029018.13) transcript is recommended as the knockout region. The region contains 1202bp coding sequence. Knock out the region will result in disruption of protein function. In this project we use CRISPR/Cas9 technology to modify *Zbp1* gene. The brief process is as follows: sgRNA was transcribed in vitro, donor vector was constructed. Cas9, sgRNA and Donor were microinjected into the fertilized eggs of C57BL/6JGpt mice. Fertilized eggs were transplanted to obtain positive F0 mice which were confirmed by PCR and sequencing. A stable F1 generation mouse model was obtained by mating positive F0 generation mice with C57BL/6JGpt mice. PCR for genotyping was performed with primers: 5’-GGAGGATTGCTATGAGTTCCAGG-3’ (sense) and 5’- CCTGATACAGCAGGAGTCCTGAA-3’ (antisense), which yielded one band with 392 bp products for *Zbp1^fl/fl^*, two bands with 392 bp and 288 bp products for *Zbp1^fl/+^* and one band with 288 bp products for WT.

*Zbp1* conditional knockin mouse model at the locus of ROSA26 was created by GemPharmatech Co., Ltd. (Nanjing, Jiangsu, China) using CRISPR/Cas-mediated genome engineering. The compound promoter, CAG, is a ubiquitously active promoter, the length is about 1.7kb. Rosa26, located on mouse chromosome 6, is a safe site for foreign gene insertion. The foreign gene integrated into this site can be expressed stably and efficiently without destroying the function of endogenous gene. A construct containing the loxP-floxed cassette with *Zbp1* gene driving by CAG promoter was inserted into the Gt(ROSA)26S or locus via homologous recombination. The floxed Stop cassette contains a strong tpA stop sequence which prevents the transcription of *Zbp1*. This entire targeting vector was inserted between exons 1 and 2 of the G(ROSA)26Sor locus. CRISPR/Cas9 system and donor were microinjected into the fertilized eggs of C57BL/6JGpt mice, and obtained positive F0 generation mice. The F0 positive mice were mated with C57BL/6JGpt mice and the pups will be genotyped by PCR with primers: F1: 5’- GGACTGAGCAAGCAAGATTACAAGG-3’ (sense) and R: 5’-TCGGGTGAGCATGTCTTTAATCT-3’ (antisense), F2: 5’- CCCAAAGTCGCTCTGAGTTGTTA-3’ (sense) and R: 5’-TCGGGTGAGCATGTCTTTAATCT-3’ (antisense), which yielded one band with 519 bp products for *Zbp1^TG/TG^*, two bands with 519 bp and 479 bp for *Zbp1^TG/+^* and one band with 479 bp for WT.

*Myh6*-Cre^ERT2^ mice were obtained from GemPharmatech Co., Ltd. (Nanjing, Jiangsu, China). *Zbp1^fl/f^,* and *Zbp1^TG/TG^ mice* were bred with *Myh6*-Cre^ERT2^ mice to achieve CM-specific *Zbp1* knockout and *Zbp1* overexpression. Cre expression was induced by daily intraperitoneal injections of 50 mg/kg tamoxifen (T5648, Sigma Aldrich) for 5 days. The control cohort of *Myh6*-Cre^ERT2^ mice received an equivalent dosage of tamoxifen administration for standardization purposes.

*Lyz2^Cre^* mice were obtained from Cyagen Biosciences Inc (Guangzhou, Guangdong, China). *Zbp1^fl/fl^* mice were bred with *Lyz2^Cre^* mice to achieve myeloid-specific *Zbp1* knockout. cGAS-deficiency mice (*cGAS^-/-^*, Stock No. 026554) were purchased from The Jackson Laboratory (Bar Harbor, ME). Sting-deficiency mice (*Sting^-/-^*, Stock No. T012747) were purchased from GemPharmatech Co., Ltd. (Nanjing, Jiangsu, China).

**Microscopic imaging of cell death**

PI (Invitrogen, P3566, 1:1000 dilution) and Hoechst (Beyotime, C1028, 1:100 dilution) dyes were added to freshly isolated hypoxic cardiomyocytes under light-proof conditions. The BioTek live cell workstation was activated along with the gas valve, maintaining a temperature of 37°C, and filled with a gas mixture comprising 21% O2, 5% CO2, and 74% N2. Subsequently, the 6-well plate containing the cardiomyocytes was positioned within the live cell workstation. Following adjustment of focus and setting of photographic parameters (including capturing images from 6 random fields of view in each well at 30-minute intervals), the workstation was initiated. The system autonomously acquired images of cardiomyocyte status and PI and Hoechst fluorescence at specified time points as per the predetermined program.

**Human Induced Pluripotent Stem Cell (iPSC) Culture and Differentiation into Cardiomyocytes**

Commercially human iPSC-CMs were purchased from Nanjing Cosmos Biotechnology Co., Ltd. Briefly, human iPSCs were maintained on Matrigel-coated culture plates (Corning, 354230) in E8 medium (Gibco, A15169). Cells at passages P20–P40 were cultured with E8 medium lacking Y-27632 (ROCK inhibitor; Tocris, 1254), and medium changes were performed daily thereafter. Cardiomyocyte differentiation was initiated once the iPSC cultures reached over 95% confluency. Following differentiation, maturation, and purification, the resulting cardiomyocytes exhibited synchronous contractile activity and can be used for subsequent experiments. ZBP1 specific siRNA (si-ZBP1) and negative control siRNA sequences (NC) were transfected using Lipofectamine® RNAiMAX Reagent (Invitrogen, 13778075). The target sequences of ZBP1 siRNA were: ACAGGTTTCTCAAAGACAATG.

**Hypoxia/reoxygenation injury of Adult Mouse Cardiomyocytes**

Adult mouse ventricular cardiomyocytes were isolated and seeded onto laminin-coated Petri dishes, where they were allowed to adhere in a standard cell culture incubator for 24 hours. Subsequently, the cardiomyocytes were transferred to ischemic buffer (118 mmol/L NaCl, 24 mmol/L NaHCO3, 1.0 mmol/L NaH2PO4, 2.5 mmol/L CaCl2-2H2O, 1.2 mmol/L MgCl2, 20 mmol/L sodium lactate, 16 mmol/L KCl, and 10 mmol/L 2-deoxyglucose; pH adjusted to 6.2) and subjected to hypoxic conditions (1% O2, 94% N2, 5% CO2) for 30 minutes within a hypoxic chamber. Following hypoxia, the ischemic buffer was replaced with fresh medium, and the cells were returned to the standard cell culture incubator for the specified reoxygenation period. After reoxygenation, both the cells and their supernatants were collected separately for further analysis.

**Lactate dehydrogenase activity detection assay**

The activity of lactate dehydrogenase (LDH) in the culture supernatant of cardiomyocytes and the serum of mice subjected to various treatments was assessed utilizing an LDH detection kit (Beyotime, China, C1028), following the manufacturer's guidelines. LDH activity was quantified by measuring absorbance at 450 nm using a microplate reader (Bio Tek, Richmond, VA, USA).

**Caspase-3 activity assay**

Caspase-3 activity was assessed following the standard manufacturer’s instructions (Beyotime, China, C1116). Briefly, cells were collected and lysed on ice for 15 minutes. Protein concentration was quantified using the Bradford Protein Assay Kit (Beyotime, China, P0006) and normalized to 1-3 mg/ml. The reactants were incubated at 37°C for 2 hours, and absorbance at 405 nm was measured using a microplate reader (Bio Tek, Richmond, VA, USA).

**Enzyme-linked immunosorbent assay (ELISA)**

Cell culture supernatants were collected and centrifuged at 3000 rpm for 20 minutes to remove debris. The IL-18 expression levels in the cardiomyocyte culture supernatant were quantified using ELISA kits (R&D Systems, USA, 7625) according to the manufacturer’s instructions.

**Histopathology Analysis**

The hearts of the mice underwent perfusion with cold PBS and subsequent harvesting. Following fixation in 4% paraformaldehyde for 48 hours, the hearts were embedded in paraffin. Transverse sections of the hearts were then obtained at 4 μm intervals, followed by dewaxing and hydration of the slides. Subsequently, the slides were subjected to incubation with Masson dye solution set (Servicebio, G1006), and images were acquired and analyzed via microscopic inspection. The fibrotic area within each field was quantified utilizing Image-Pro Plus software. For quantification purposes, the total fibrosis was averaged from 2-3 fields per section and 3 heart sections per mouse. Each experimental group consisted of 10 mice.

For immunohistochemistry, the slides were incubated with the primary antibody at 4°C overnight. The following day, the slides were incubated with a horseradish peroxidase (HRP)-conjugated secondary antibody at room temperature for 50 minutes. Subsequently, DAB color development solution was applied, followed by hematoxylin counterstaining of the nuclei. The hematoxylin-stained nuclei appear blue, while the DAB-positive signal is brown-yellow. For quantification, the total DAB-positive signal was averaged from 2-3 fields per section and 3 heart sections per heart.

**Immunofluorescence Staining**

Mouse hearts were embedded in OCT compound, and serial sections were transversely obtained at 6 μm intervals. Adult mouse cardiomyocytes and heart sections underwent fixation in 4% paraformaldehyde, followed by blocking with 3% BSA and permeabilization with 0.3% Triton-X100. Subsequently, the cells and heart sections were incubated with primary antibodies against α-actinin (abcam, ab68194, 1:200 dilution), Zbp1 (Adipogen, AG-20B-0010-C100, 1:100), Vimentin (abcam, ab92547, 1:200), CD31 (R&D Systems, AF3628, 1:200), CD45 (abcam, ab10558, 1:200), RIPK3 (Prosci, 2283, 1:100), Caspase 8 (CST, 4790, 1:100) and Caspase 6 (CST, 9762, 1:100) according to the specified groups, incubated at 4 °C overnight. The following day, the cells and heart sections were incubated with Alexa Fluor™ Plus 488 Donkey anti-Mouse IgG (H+L) Highly Cross-Adsorbed Secondary Antibody (Invitrogen, A32766, 1:500 dilution) and Alexa Fluor™ Plus 647 Goat anti-Rabbit IgG (H+L) Highly Cross-Adsorbed Secondary Antibody (Invitrogen, A32733, 1:500 dilution) at room temperature for 1 hour, protected from light. After washing three times for 5 minutes, 4,6-Diamidino-2-phenylindole (DAPI) (Invitrogen, D21490) was utilized to stain the nuclei for an additional 10 minutes in darkness. Immunofluorescence staining was captured using a Zeiss fluorescence microscope, and the fluorescence intensity was quantified using Image-Pro Plus software (version 6.0).

TUNEL staining was conducted using the One Step TUNEL Apoptosis Assay Kit (Beyotime, Shanghai, China) following the manufacturer's protocol. In brief, the samples underwent fixation and subsequent staining with the TUNEL reagent. After counterstaining with DAPI, the samples were visualized using a Zeiss fluorescence microscope.

**Evans blue dye (EBD) uptake assay**

The EBD (Sigma, E2129) solution was dissolved in PBS and subsequently sterilized by filtration through a 0.22-μm filter. Mice received an intraperitoneal injection of 10 mg/ml Evans Blue dye (100 mg/kg of mouse body weight) 24 hours before sacrifice. Then the mice were anesthetized, and their hearts were excised, washed with saline, and processed for cryosectioning using OCT embedding as described previously, with the entire process conducted under light protection. Following immunofluorescence staining with α-actinin (Sigma-Aldrich, A5044, 1:200 dilution) or FITC-conjugated wheat germ agglutinin (WGA, W11261, Invitrogen), cardiomyocytes were visualized and imaged under a Zeiss fluorescence microscope. EBD signal was detected using a 647nm excitation wavelength, and fluorescence intensity was quantified using Image-Pro Plus software (version 6.0).

**Adenovirus Construction and Infection**

For adenovirus construction, the entire coding regions of ZBP1 were inserted into the pAdeasy-1 vector (Hanbio Co. Ltd, Shanghai, China). DNA sequences encoding shRNAs for RIPK3 (Ad-sh-RIPK3, 5’-CCTCAGATTCCACATACTTTA-3’), caspase-6 (Ad-sh-caspase-6, 5’-CGATTGCTTCATCTGTGTCTT -3’), caspase-8 (Ad-sh-caspase-8, 5’-GGATGTTGGAGGAAGGCAATC-3’), and a scrambled control were cloned into the pAdeasy-1 vector. The recombinant adenoviruses were generated by recombnating the modified pAdeasy-1 plasmids and shuttle plasmid plasmids in E. coli BJ5183 and amplificated in 293A cells by using LipofectamineTM 3000 Reagent (Invitrogen, L3000001). AMCMs were infected with the indicated adenovirus particles at a multiplicity of infection (MOI) of 100 for 12h before replacing the medium. 72h after infection, cells were used for bio-analysis. All the procedures were conducted in a biosafety environment.

**Western Blotting**

Left ventricular tissue homogenates and cell lysates were prepared in RIPA lysis buffer (P0013, Beyotime, China) supplemented with protease inhibitor cocktail (Thermo, 78443, diluted 1:100) on ice. Following centrifugation at 13,500 g at 4 °C for 15 minutes, supernatant fractions were collected, and the protein concentration was determined using the manufacturer's protocol (P0010, Beyotime, China). Equal amounts of proteins were denatured and separated in 10-15% SDS-polyacrylamide gels. Subsequently, proteins were transferred onto poly vinylidene fluoride (PVDF) membranes (Merck, #ISEQ00010). The membranes were blocked with 5% non-fat milk for 1h at room temperature and then incubated with primary antibodies at indicated dilutions at 4°C overnight (Supplementary Table 2). Horseradish peroxidase (HRP)-conjugated anti-rabbit or anti-mouse secondary antibodies were used at a 1:4000 dilution for 1 hour at room temperature to detect the primary antibodies. Protein bands were visualized using an Electrochemiluminescence (ECL) substrate (Thermo, #32132) and a ChemiDoc™ Imaging System (Bio-Rad, CA, USA). Band densities were quantified after normalization using NIH ImageJ software (version 1.50i, USA).

**Quantitative Real-Time PCR**

Total RNA from cultured cells or heart tissues was isolated and collected using RNAiso Plus (Takara, 9109) following the manufacturer’s protocol. The concentration and purity of RNA were assessed using a Nanodrop spectrophotometer. RNA samples with a A260/A280 ratio between 1.8 and 2.0 were utilized for subsequent reverse transcription using PrimeScript RT Master Mix (Takara, #RR036A). Quantitative real-time polymerase chain reaction (qRT-PCR) was performed with SYBR Green dye (Takara, #RR820A) and specific gene primers (Supplementary Table 3) on a CFX96 real-time PCR System (Bio-Rad Laboratories, Inc., CA, USA) under the recommended thermal cycling conditions. The expression levels of target genes were normalized to ACTB using the standard 2^−ΔΔCT^ method.

**Co-immunoprecipitation**

The co-immunoprecipitation procedure followed the guidelines provided in the Pierce Classic IP Kit (Thermo Scientific™, 26146) with slight adaptations. Initially, cultured cells underwent PBS washes and were lysed with ice-cold IP Lysis/Wash Buffer. Following centrifugation at 13,000 g for 10 minutes, the supernatant was transferred to a new tube for protein concentration determination. Subsequently, lysates were preincubated with Control Agarose Resin slurry for 1 hour and then subjected to overnight incubation at 4 ℃ with rotation with either DYKDDDDK Tag antibody (CST, 14793), RIPK3 antibody (Prosci, 2283) or Caspase 6 antibody (CST, 9762). The following day, Pierce Protein A/G Agarose was introduced to the lysates and incubated for 1 hour at 4 °C to facilitate agarose-antibody-antigen complex formation, which was then collected using a Pierce spin column. Following three washes in IP Lysis/Wash Buffer and one wash in 1X Conditioning Buffer, proteins were dissociated from the beads by resuspending in 50 μL of 2× loading buffer and subsequent boiling at 95 °C for 10 minutes. The resulting samples were subjected to immunoblotting. To prevent interference from heavy and light chains of IgGs, the Clean-Blot IP Detection Kit (Thermo Scientific™, 21232) was employed to detect target proteins.

**RNA-Sequencing and Data Analysis**

Total RNA was extracted from cardiac tissue or cardiomyocytes samples obtained from mice, using the miRNA mirVana Isolation Kit (Ambion) following the manufacturer's protocol. RNA integrity was assessed using an Agilent 2100 Bioanalyzer (Agilent Technologies, Santa Clara, CA), with samples having an RNA Integrity Number (RIN) of 7 or higher deemed suitable for further analysis. Library construction was carried out using the TruSeq Stranded mRNA LTSample Prep Kit from Illumina (San Diego, CA, USA), adhering to the manufacturer's instructions. Subsequently, these libraries underwent sequencing on the Illumina sequencing platform (HiSeqTM 2500 or Illumina HiSeq X Ten) to generate 125bp/150bp paired-end reads. Raw data (raw reads) were processed using Trimmomatic to obtain clean reads, which involved the removal of reads containing poly-N and low-quality reads, before mapping the clean reads to the reference genome using hisat2. Cufflinks were employed to obtain the Fragments Per Kilobase of transcript per Million mapped reads (FPKM) value for each gene, and read counts were calculated using htseq-count. The DESeq (2012) R package functions estimateSizeFactors and nbinomTest were applied to identify Differential Expressed Genes (DEGs), with a predefined threshold of FDR-corrected q-value < 0.05 and foldChange < 1/1.5 or foldChange >1.5. Hierarchical cluster analysis and Principal Component Analysis (PCA) were performed using the R packages "pheatmap" and "factoextra," respectively. Coexpression analysis and GO enrichment analysis of DEGs were performed using R package “clusterProfiler”, "org.Mm.eg.db" and “ggplot2”.

**Protein Structure Preparation and Virtual Screening**

The three-dimensional structure of Mouse ZBP1 was retrieved from the AlphaFold Protein Structure Database (AlphaFold ID: AF-Q9QY24-F1). Structural preparation included the addition of hydrogen atoms, optimization of hydrogen-bonding networks, and restrained energy minimization using the OPLS2005 force field, with a root mean square deviation (RMSD) convergence threshold set at 0.30 Å. Potential ligand-binding sites on the prepared Mouse ZBP1 structure were predicted using the SiteMap module (Schrödinger). Three commercially available compound libraries from MCE, comprising 25,300, 80,000, and 200,000 compounds respectively, were employed for virtual screening. The two-dimensional structures of these compounds were processed using the LigPrep module (Schrödinger) to generate three-dimensional conformations suitable for molecular docking. Virtual screening against the Mouse ZBP1 Site4 grid was performed using the Virtual Screening Workflow (VSW) module within Schrödinger Maestro 12.8, employing the Glide docking engine.

**Surface Plasmon Resonance (SPR) Analysis**

The CMS sensor chip (BR100530, Cytiva) was installed in the instrument with the label-side facing upwards, following the directional arrow on the chip. Flow cell 2 (FC2) was activated at 10 µL/min using a 1:1 mixture of 0.4 M EDC (1-Ethyl-3-(3-dimethylaminopropyl) carbodiimide) and 0.1 M NHS (N-Hydroxysuccinimide) from the Amine Coupling Kit (BR100050, Cytiva). Mouse ZBP1 protein was diluted to a final concentration of 50 µg/mL in 10 mM sodium acetate buffer and injected over the activated FC2 surface at 10 µL/min until the desired immobilization level was achieved. The immobilization curve was recorded in real-time. For single-concentration surface plasmon resonance (SPR) analysis, test compounds were sequentially injected over both the reference (FC1) and ZBP1-immobilized (FC2) surfaces at a flow rate of 10 µL/min for 150 seconds (association phase), followed by a 300-second dissociation and regeneration phase using 10 mM glycine hydrochloride (pH 2.0). Real-time sensorgrams were recorded. For concentration-dependent analysis, MSB was serially diluted into multiple concentrations and injected using a 96-well format over both FC1 and FC2 at 30 µL/min for 150 seconds, followed by a 300-second regeneration step in 10 mM glycine hydrochloride (pH 2.0). Binding kinetics were analyzed using a global fit to a 1:1 Langmuir binding model in Biacore Insight Evaluation Software (Cytiva, Marlborough, MA, USA).

**Figure. S1. Identify the dynamic molecular changes following myocardial I/R injury.**

Temporal expression trends based on clustering of coexpressed differentially expressed genes (DEGs) during I/R. Clusters 1-3: Genes were significantly upregulated from reperfusion 0 to 24 hours and either significantly upregulated (Cluster 1), significantly downregulated (Cluster 2), or unchanged (Cluster 3) at 0 hour of reperfusion (after 30 minutes of ischemia) compared with the sham group. Clusters 4-6: Genes were significantly downregulated from reperfusion 0 to 24 hours and either significantly upregulated (Cluster 4), significantly downregulated (Cluster 5), or unchanged (Cluster 6) at 0 hour of reperfusion (after 30 minutes of ischemia) compared with the sham group. Each data point represents the relative gene expression level at a given time point of I/R in logarithm base 10 scale. The color gradient of each line indicates the magnitude of fold change for the corresponding gene, ranging from low (blue) to high (purple-red). GO enrichment analysis showed significant enrichments of the DEGs in each cluster.

**Figure. S2. ZBP1 expression was not changed in non-cardiomyocytes after I/R injury.**

**a.** Quantitative real time polymerase chain reaction (qRT-PCR) analyses of Zbp1 mRNA levels in cardiac fibroblasts isolated from WT mice 24-hours post-I/R or Sham surgery (n=6). **b.** Western blot of ZBP1 and Vimentin in cardiac fibroblasts isolated from WT mice 24-hours post-I/R or Sham surgery (n=3). **c.** Representative immunofluorescence staining of DAPI (blue), ZBP1 (green) and Vimentin (red) in myocardium of WT mice 24-hours post-I/R or Sham surgery (scale bar = 10 μm). **d.** Quantitative real time polymerase chain reaction (qRT-PCR) analyses of Zbp1 mRNA levels in endothelial cells isolated from WT mice 24-hours post-I/R or Sham surgery (n=6). **e.** Western blot of ZBP1 and CD31 in endothelial cells isolated from WT mice 24-hours post-I/R or Sham surgery (n=3). **f.** Representative immunofluorescence staining of DAPI (blue), ZBP1 (green) and CD31 (red) in myocardium of WT mice 24-hours post-I/R or Sham surgery (scale bar = 10 μm). **g.** Quantitative real time polymerase chain reaction (qRT-PCR) analyses of Zbp1 mRNA levels in immune cells isolated from WT mice 24-hours post-I/R or Sham surgery (n=6). **h.** Western blot of ZBP1 and CD45 in immune cells isolated from WT mice 24-hours post-I/R or Sham surgery (n=3). **i.** Representative immunofluorescence staining of DAPI (blue), ZBP1 (green) and CD45 (red) in myocardium of WT mice 24-hours post-I/R or Sham surgery (scale bar = 10 μm). For all statistical plots, the data are presented as mean ± SEM. a, d and g by two-tailed unpaired Student’s t-test.

**Figure. S3. ZBP1 expression was not altered by the deficiency of either cGAS or STING.**

**a.** Western blot of ZBP1 in cardiomyocytes isolated from WT or *cGAS*^-/-^ mice that underwent H/R for 0.5/24h. **b.** qRT-PCR analyses of Zbp1, Ifit1 and Ifi44 mRNA levels in cardiomyocytes isolated from WT or *cGAS*^-/-^ mice that underwent H/R for 0.5/24h (n=4). **c.** Western blot of ZBP1 in cardiomyocytes isolated from WT or *Sting*^-/-^ mice that underwent H/R for 0.5/24h. **d.** qRT-PCR analyses of Zbp1, Ifit1 and Ifi44 mRNA levels in cardiomyocytes isolated from WT or *Sting*^-/-^ mice that underwent H/R for 0.5/24h (n=4). For all statistical plots, the data are presented as mean ± SEM. b and d by Welch’s ANOVA with Dunnett's T3 multiple comparison test.

**Figure. S4. The construction of cardiomyocyte specific Zbp1 knockout mice.**

**a.** Schematic diagram for generating cardiomyocyte-specific tamoxifen-inducible *Zbp1* knockout mice. **b.** The DNA agarose gel electrophoresis showing genotyping results of *Zbp1*-cKO mice. **c.** qRT-PCR analyses of Zbp1 mRNA levels in cardiomyocytes and non-cardiomyocytes isolated from WT mice (n=4). **d.** qRT-PCR analyses of Zbp1 mRNA levels in cardiomyocytes isolated from *Zbp1^fl/fl^Myh6*^+^ and *Myh6*^+^ mice (n=4). **e.** qRT-PCR analyses of Zbp1 mRNA levels in non-cardiomyocytes isolated from *Zbp1^fl/fl^Myh6*^+^ and *Myh6*^+^ mice (n=4). **f.** Immunofluorescence and quantification of ZBP1 (red) in myocardium of *Zbp1^fl/fl^Myh6*^+^ and *Myh6*^+^ mice (n=4; scale bar=500 μm in upper panel and 20 μm in lower panel). For all statistical plots, the data are presented as mean ± SEM. c and e-f by two-tailed unpaired Student’s t-test. d by Welch’s t-test.

**Figure. S5. *Zbp1* Deficiency does not exert significant effects on inflammatory responses.**

**a.** Western blot of phosphorylated-STING (p-STING) and STING protein levels in cardiomyocytes isolated from *Myh6*^+^ and *Zbp1^fl/fl^Myh6*^+^ that underwent H/R for 0.5/1h. **b.** qRT-PCR analyses of Ifit1 and Ifi44 mRNA levels in cardiomyocytes isolated from *Myh6*^+^ and *Zbp1^fl/fl^Myh6*^+^ that underwent H/R for 0.5/1h. **c.** Western blot of phosphorylated-NFκB (p-NFκB) and NFκB protein levels in cardiomyocytes isolated from *Myh6*^+^ and *Zbp1^fl/fl^Myh6*^+^ that underwent H/R for 0.5/1h. **d.** qRT-PCR analyses of IL-1β and IL-6 mRNA levels in cardiomyocytes isolated from *Myh6*^+^ and *Zbp1^fl/fl^Myh6*^+^ that underwent H/R for 0.5/1h. For all statistical plots, the data are presented as mean ± SEM. b and d by Welch’s ANOVA with Dunnett's T3 multiple comparison test.

**Figure. S6. ZBP1 deficiency protects human iPSC-derived cardiomyocytes against H/R stress**

**a.** Immunofluorescence of ZBP1 in human iPSC-derived cardiomyocytes with or without H/R treatment for 12/24h (scale bar = 20μm). **b.** qRT-PCR analyses of ZBP1 mRNA levels in human iPSC-derived cardiomyocytes with or without H/R treatment for 12/24h (n=4). **c.** Propidium iodide (PI) and Hoechst staining of human iPSC-derived cardiomyocytes transfected with si-NC or si-ZBP1 with or without H/R treatment for 12/24h (n=4, scale bar = 20μm). **d.** LDH (lactate dehydrogenase) release of human iPSC-derived cardiomyocytes transfected with si-NC or si-ZBP1 with or without H/R treatment for 12/24h (n=4). **e.** Caspase-3 activity of human iPSC-derived cardiomyocytes transfected with si-NC or si-ZBP1 with or without H/R treatment for 12/24h (n=4). For all statistical plots, the data are presented as mean ± SEM. b by Welch’s t-test. c by Welch’s ANOVA with Dunnett's T3 multiple comparison test. d-e by two-way ANOVA with Bonferroni multiple comparison test

**Figure. S7. Cardiomyocyte-specific *Zbp1* deficiency ameliorates I/R-induced cardiac dysfunction**

**a.** M-mode echocardiography of *Myh6^+^* and *Zbp1^fl/fl^Myh6^+^* mice 28 days post-I/R injury. **b-d.** left ventricular EF (**b**), FS (**c**) and LVID (**d**) assessed by echocardiography in *Myh6^+^* and *Zbp1^fl/fl^Myh6^+^* mice 28 days post-I/R injury (n=10). **e.** Representative photographs and quantitative analysis of Masson staining of heart sections from *Myh6^+^* and *Zbp1^fl/fl^Myh6^+^* mice 28 days post-I/R injury (n=10, scale bar = 500 μm). **f.** qRT-PCR analyses of mRNA levels of Nppb and Col1a1 in heart samples from *Myh6^+^* and *Zbp1^fl/fl^Myh6^+^* mice 28 days post-I/R injury (n=4). For all statistical plots, the data are presented as mean ± SEM. b-f by two-way ANOVA with Bonferroni multiple comparison test. EF, ejection fraction; FS, fractional shortening; LVID_s_, left ventricular internal diameter at end-systole; LVID_d_, left ventricular internal diameter at end-diastole.

**Figure. S8. Myeloid-specific *Zbp1* deficiency has minimal impact on I/R induced cardiomyocyte death**

**a.** Schematic diagram for generating myeloid -specific *Zbp1* knockout mice. **b.** qRT-PCR analyses of Zbp1 mRNA levels in bone marrow cells isolated from *Zbp1^fl/fl^Lyz2*^+^ and *Lyz2*^+^ mice (n=6). **c.** The serum levels of lactate dehydrogenase (LDH, U/L) of *Zbp1^fl/fl^Lyz2*^+^ and *Lyz2*^+^ mice 24 hours after Sham or I/R surgery (n=8). **d.** Representative images of heart sections stained by Evans blue and TTC at 24 h after myocardial-ischemia reperfusion injury of *Zbp1^fl/fl^Lyz2*^+^ and *Lyz2*^+^ mice. The ratio of area at risk (AAR) to left ventricle (LV) and infarct area (IA) to AAR were calculated based on Evans blue and TTC staining (n=6). For all statistical plots, the data are presented as mean ± SEM. b and d by two-tailed unpaired Student’s t-test. c by two-way ANOVA with Bonferroni multiple comparison test.

**Figure. S9. The construction of cardiomyocyte specific Zbp1 overexpression mice.**

**a.** Schematic diagram for generating cardiomyocyte-specific tamoxifen-inducible Zbp1 overexpression mice. Zbp1^TG^, Zbp1 conditional transgene mice. **b.** The DNA agarose gel electrophoresis showing genotyping results of Zbp1 overexpression mice. **c.** qRT-PCR analyses of mRNA levels of Zbp1 in cardiomyocytes isolated from *Zbp1^TG^Myh6*^+^ and *Myh6*^+^ mice (n=4). **d.** Western blot and quantification of ZBP1 in cardiomyocytes isolated from *Zbp1^TG^Myh6*^+^ and *Myh6*^+^ mice (n=3). **e.** Immunofluorescence of DAPI (blue), ZBP1 (green) and α-actinin (red) in myocardium of *Zbp1^TG^Myh6*^+^ and *Myh6*^+^ mice (scale bar=20 μm in left panel and 10 μm in right panel). For all statistical plots, the data are presented as mean ± SEM. c by two-tailed unpaired Student’s t-test. d by Welch's t-test.

**Figure. S10. *Zbp1* overexpression induces effects associated with cell death.**

**a.** Principal component analysis (PCA) showing group distribution based on RNA-seq analyses of cardiomyocytes isolated from *Myh6*^+^ and *Zbp1^TG^Myh6^+^* mice 8 weeks post tamoxifen injection. **b.** Volcano plot showing the gene expression of significantly upregulated and downregulated genes based on RNA-seq data. **c-d.** GO term enrichment analysis of upregulated (**c**) and downregulated (**d**) genes in cardiomyocytes isolated from *Myh6*^+^ and *Zbp1^TG^Myh6^+^* mice show the top 10 biological process based on RNA-seq data.

**Figure. S11. ZBP1 drive cardiomyocyte PANoptosis through a noncanonical pathway.**

**a.** Western blot and quantification of total caspase 8 (T-Caspase 8) and cleaved caspase 8 (Cl-Caspase 8) protein levels in heart samples of *Myh6*^+^ and *Zbp1^fl/fl^Myh6*^+^ mice 24 hours after I/R surgery (n=4). **b.** Western blot and quantification of phosphorylated-RIPK1 (p-RIPK1) and RIPK1 protein levels in heart samples of *Myh6*^+^ and *Zbp1^fl/fl^Myh6*^+^ mice 24 hours after I/R surgery (n=4). For all statistical plots, the data are presented as mean ± SEM. a-b by two-way ANOVA with Bonferroni multiple comparison test.

**Figure. S12. ZBP1 overexpression was sufficient to induce cardiomyocyte death.**

**a.** Representative TUNEL staining and relative quantification in adult cardiomyocytes infected with adenovirus expressing ZBP1 or NC for 72h (n=6; scale bar = 50μm). **b.** Microscopic images and quantification of adult cardiomyocytes infected with adenovirus expressing ZBP1 or NC were treated with propidium iodide (PI) and Hoechst staining for 72h (n=6; scale bar = 50μm). **c.** Caspase-3 activity of adult cardiomyocytes infected with adenovirus expressing ZBP1 or NC and then treated with LDC7559 (1uM), Nec-1 (1uM), GSK’872 (3uM), Z-VAD-FMK (5uM) or Vehicle (n=6). **d.** LDH (lactate dehydrogenase) release in the supernatants of adult cardiomyocytes infected with adenovirus expressing ZBP1 or NC and then treated with LDC7559 (1uM), Nec-1 (1uM), GSK’872 (3uM), Z-VAD-FMK (5uM) or Vehicle (n=6). **e.** IL (interleukin)-18 release in the supernatants of adult cardiomyocytes infected with adenovirus expressing ZBP1 or NC and then treated with LDC7559 (1uM), Nec-1 (1uM), GSK’872 (3uM), Z-VAD-FMK (5uM) or Vehicle (n=6). For all statistical plots, the data are presented as mean ± SEM. a-b by Welch's t-test. c-e by one-way ANOVA with Bonferroni multiple comparison test.

**Figure. S13. CAMKII activation was regulated by ZBP1**

**a.** Coimmunoprecipitation and Western blot assays of RIPK3 and CAMKII interaction in cardiomyocytes infected with adenovirus expressing ZBP1 or NC. **b.** Western blot and quantification of phosphorylated-CAMKII (p-CAMKII) and CAMKII protein levels in cardiomyocytes isolated from *Myh6*^+^ and *Zbp1^TG^Myh6^+^* mice 8 weeks post tamoxifen injection (n=6). **c.** Western blot and quantification of phosphorylated-CAMKII (p-CAMKII) and CAMKII protein levels in heart samples of *Myh6*^+^ and *Zbp1^fl/fl^Myh6*^+^ mice 24 hours after I/R surgery (n=4). For all statistical plots, the data are presented as mean ± SEM. b by two-tailed unpaired Student’s t-test. c by two-way ANOVA with Bonferroni multiple comparison test.

**Figure. S14. Cell-death inhibitors ameliorated cardiac remodeling induced by *Zbp1* overexpression**

**a.** M-mode echocardiography of *Myh6^+^* and *Zbp1^TG^Myh6^+^* mice treated with Nec-1 (1mg/kg), GSK’872 (5mg/kg), Z-VAD-FMK (2mg/kg) or Vehicle control. **b-c.** left ventricular EF (**b**) and FS (**c**) assessed by echocardiography in *Myh6^+^* and *Zbp1^TG^Myh6^+^* mice of indicated groups (n=10). **d.** Representative photographs and quantitative analysis of Masson staining of heart sections from *Myh6^+^* and *Zbp1^TG^Myh6^+^* mice of indicated groups (n=10; scale bar = 500μm). **e.** Representative photographs and quantitative analysis of myocardial Evans blue dye (EBD) uptake and WGA (wheat germ agglutinin) staining in *Myh6^+^* and *Zbp1^TG^Myh6^+^* mice of indicated groups (n=10; scale bar = 20μm). **f.** qRT-PCR analyses of Ifit1 and Ifi44 mRNA levels in heart tissues from *Myh6^+^* and *Zbp1^TG^Myh6^+^* mice of indicated groups (n=6). For all statistical plots, the data are presented as mean ± SEM. b-d by one-way ANOVA with Bonferroni multiple comparison test. e-f by Welch’s ANOVA with Dunnett's T3 multiple comparison test. EF, ejection fraction; FS, fractional shortening.

**Figure. S15. MSB treatment protects cardiomyocytes against H/R stress**

**a.** Propidium iodide (PI) and Hoechst staining of adult cardiomyocytes treated with MSB (1μM) or Vehicle with or without H/R treatment for 0.5/24h (n=4; scale bar = 50μm). **b.** LDH (lactate dehydrogenase) release of adult cardiomyocytes treated with MSB (1μM) or Vehicle with or without H/R treatment for 0.5/24h (n=4). **c.** Caspase-3 activity of adult cardiomyocytes treated with MSB (1μM) or Vehicle with or without H/R treatment for 0.5/24h (n=4). For all statistical plots, the data are presented as mean ± SEM. a by Welch’s ANOVA with Dunnett's T3 multiple comparison test. b-c by two-way ANOVA with Bonferroni multiple comparison test.

**Table S1.** Clinical characteristics of patients with Normal or ICM.

Normal (n=6)	ICM (n=8)	p

Age of diagnosis (years)	53.47±3.79	60.13±4.15	0.010

Female n (%)	3 (50%)	4 (50%)	>0.999

BMI	22.47±2.88	23.56±2.13	0.430

Echocardiographic parameters

LVEDD (mm)	42.12±4.14	63.65±6.48	<0.001

LVESD (mm)	33.27±3.23	41.67±6.34	0.012

LVEF (%)	59.11±4.58	27.34±5.45	<0.001

Diseases

Hypertension (%)	1 (17%)	5 (63%)	0.138

Atrial fibrillation (%)	0 (0%)	2 (25%)	0.473

Valvular heart disease (%)	0 (0%)	1 (12.5%)	>0.999

Diabetes (%)	2 (33%)	4 (50%)	0.627

Hyperlipemia (%)	2 (33%)	6 (75%)	0.227

Medical therapy prior to the surgery

Anticoagulant n (%)	1 (17%)	3 (38%)	0.580

Antiplatelet n (%)	2 (33%)	8 (100%)	0.015

Statin n (%)	2 (33%)	7 (88%)	0.091

Diuretics n (%)	0 (0%)	5 (63%)	0.031

β-blockers n (%)	1 (17%)	5 (63%)	0.138

ACEI/ARB n (%)	1 (17%)	6 (75%)	0.103

Data are expressed as mean ± SD. Data of Ages, BMI and Echocardiographic parameters were analyzed using two-tailed unpaired Student’s t-test. Others were analyzed using Fisher exact test. ICM Ischemic cardiomyopathy, BMI body mass index, LVEDD left ventricular end diastolic diameter, LVESD left ventricular end systolic diameter, LVEF left ventricular ejection fraction, ACEI angiotensin-converting enzyme inhibitor, and ARB angiotensin receptor blocker

**Table S2.** The information of antibodies.

| Name | Vender | Cat |
| --- | --- | --- |
| ZBP1 | Adipogen | AG-20B-0010-C100 |
| α-actinin | Abcam | ab68194 |
| α-actinin | Sigma-Aldrich | A5044 |
| Vimentin | Abcam | ab92547 |
| CD31 | R&D Systems | AF3628 |
| CD45 | Abcam | ab10558 |
| Caspase 8 | CST | 4790 |
| Cleaved Caspase 8 | Proteintech | 13423-1-AP |
| Caspase 3 | CST | 9662 |
| Cleaved Caspase 3 | CST | 9661 |
| GSDMD | NOVUS | NBP2-33422 |
| GSDMD | Abcam | ab209845 |
| p-RIPK3 | Abcam | ab195117 |
| RIPK3 | Prosci | 2283 |
| p-RIPK1 | Affinity Biosciences | AF2398 |
| RIPK1 | BD Biosciences | 610458 |
| p-CAMKII | Invitrogen | PA5-37833 |
| CAMKII | Abcam | Ab181052 |
| p-NFκB | CST | 3033 |
| NFκB | CST | 8242 |
| p-STING | CST | 72971 |
| STING | CST | 13647 |
| cGAS | CST | 31659 |
| DYKDDDDK Tag | CST | 14793 |
| Collagen I | Abcam | ab260043 |
| Myh7 | Proteintech | 22280-1-AP |
| BNP | Abcam | ab239510 |
| Ig G | CST | 2729 |
| Caspase 6 | CST | 9762 |
| NLRP3 | CST | 15101 |
| ASC | Proteintech | 10500-1-AP |
| β-actin | CST | 4970 |
| HRP-conjugated Affinipure Goat Anti-Rabbit IgG(H+L) | Proteintech | SA00001-2 |
| HRP-conjugated Affinipure Goat Anti-Mouse IgG(H+L) | Proteintech | SA00001-1 |
| HRP-conjugated Affinipure Goat Anti-Rat IgG(H+L) | Proteintech | SA00001-15 |
| Goat anti-Rabbit IgG (H+L) Alexa Fluor Plus 647 | Invitrogen | A32733 |
| Donkey anti-mouse IgG (H+L) Alexa Fluor Plus 488 | Invitrogen | A32766 |
| Donkey anti-Goat IgG (H+L) Alexa Fluor Plus 647 | Invitrogen | A32849 |

**Table S3.** Table of sequences and downstream primers for genes analyzed by qRT-PCR (m-mouse, h-human).

| Gene | Forward Primer | Reverse Primer |
| --- | --- | --- |
| Zbp1 (m) | 5’-atccctgagaactccagtgc-3’ | 5’-cgtctgtccgtcatagctca-3’ |
| Zbp1 (h) | 5’-gcagctacaattccagagacc-3’ | 5’-cctctggggaccattgtctt-3’ |
| Ifit1 (m) | 5’-agcagtcagaagacaaggcaatcac-3’ | 5’-aagcagtcagtagtttcctccaagc-3’ |
| Ifi44 (m) | 5’-ctgattacaaaagaagacatgacagac-3’ | 5’-aggcaaaaccaaagactcca-3’ |
| Il1b (m) | 5’-tccaggatgaggacatgagcac-3’ | 5’-gaacgtcacacaccagcaggtta-3’ |
| Il6 (m) | 5’-cttcttgggactgatgctggtgac-3’ | 5’-tctgttgggagtggtatcctctgtg-3’ |
| Nppb (m) | 5’-ggaagtcctagccagtctccagag-3’ | 5’-gccttggtccttcaagagctgtc-3’ |
| Col1a1 (m) | 5’-gacaggcgaacaaggtgacagag-3’ | 5’-caggagaaccaggagaaccaggag-3’ |
| β-actin (m) | 5’-tatgctctccctcacgccatcc-3’ | 5’-gtcacgcacgatttccctctcag-3’ |

**Table S4.** Biometric and cardiac parameters of *Myh6^+^* and *Zbp1^fl/fl^Myh6^+^* mice on days 28 post-IR or Sham.

| Group | *Myh6^+^*  Sham | *Zbp1^fl/fl^Myh6^+^*  Sham | *Myh6^+^*  IR | *Zbp1^fl/fl^Myh6^+^*  IR |
| --- | --- | --- | --- | --- |
| n | 10 | 10 | 10 | 10 |
| BW (g) | 24.91±0.379 | 24.11±0.668 | 24.04±0.535 | 24.52±0.721 |
| HR (beats/min) | 498±3.603 | 501±3.278 | 504±3.740 | 500±4.138 |
| EF (%) | 59.453±1.205 | 58.162±1.483 | 38.240±1.791 | 45.827±1.213 |
| FS (%) | 30.728±0.818 | 30.044±1.060 | 18.312±0.961 | 22.387±0.698 |
| LVID_d_ | 3.330±0.084 | 3.541±0.108 | 4.143±0.109 | 3.728±0.112 |
| LVID_s_ | 2.306±0.064 | 2.474±0.074 | 3.389±0.110 | 2.894±0.091 |
| IVS_d_ | 0.774±0.064 | 0.734±0.047 | 0.721±0.072 | 0.749±0.054 |
| IVS_s_ | 1.049±0.085 | 1.038±0.086 | 0.932±0.115 | 1.023±0.102 |
| LVPW_d_ | 0.888±0.085 | 0.755±0.035 | 0.793±0.032 | 0.846±0.083 |
| LVPW_s_ | 1.087±0.097 | 0.990±0.072 | 0.865±0.035 | 0.939±0.078 |

Data are expressed as mean ± SEM. Data was analyzed using two-way ANOVA followed by Bonferroni post hoc analysis. BW, body weight; HR, heart rate; EF, ejection fraction; FS, fractional shortening; LVID_d_, left ventricular internal diameter at end-diastole; LVID_s_, left ventricular internal diameter at end-systole; IVS_d_, interventricular septum thickness at end-diastole; IVS_s_, interventricular septum thickness at end-systole; LVPW_d_, left ventricular posterior wall at end-diastole; LVPW_s_, left ventricular posterior wall at end-systole.

**Table S5.** Biometric and cardiac parameters of *Myh6^+^* and *Zbp1*^TG^*Myh6*^+^ mice 8 weeks post-TAM administration.

| Group | *Myh6^+^* | *Zbp1*^TG^*Myh6^+^* |
| --- | --- | --- |
| n | 10 | 10 |
| BW | 26.71±0.551 | 26.87±0.756 |
| HR | 492±3.013 | 496±3.968 |
| EF (%) | 59.880±1.595 | 30.350±2.945 |
| FS (%) | 31.240±1.081 | 14.210±1.492 |
| LVID_d_ | 3.569±0.067 | 4.093±0.059 |
| LVID_s_ | 2.454±0.061 | 3.512±0.083 |
| IVS_d_ | 0.950±0.081 | 0.817±0.045 |
| IVS_s_ | 1.187±0.051 | 1.010±0.056 |
| LVPW_d_ | 0.769±0.042 | 0.644±0.035 |
| LVPW_s_ | 1.083±0.038 | 0.752±0.036 |

Data are expressed as mean ± SEM. Data was analyzed using two-tailed Student’s t-test. BW, body weight; HR, heart rate; EF, ejection fraction; FS, fractional shortening; LVID_d_, left ventricular internal diameter at end-diastole; LVID_s_, left ventricular internal diameter at end-systole; IVS_d_, interventricular septum thickness at end-diastole; IVS_s_, interventricular septum thickness at end-systole; LVPW_d_, left ventricular posterior wall at end-diastole; LVPW_s_, left ventricular posterior wall at end-systole.

**Table S6.** Biometric and cardiac parameters of *Myh6^+^* and *Zbp1*^TG^*Myh6*^+^ mice 8 weeks post-TAM administration.

| Group | *Myh6^+^* | *Zbp1*^TG^  *Myh6^+^*+ Vehicle | *Zbp1*^TG^  *Myh6^+^*+ Nec-1 | *Zbp1*^TG^  *Myh6^+^*+ GSK’872 | *Zbp1*^TG^  *Myh6^+^*+ zVAD | *Zbp1*^TG^  *Myh6^+^*+ GSK’872  + zVAD |
| --- | --- | --- | --- | --- | --- | --- |
| n | 10 | 10 | 10 | 10 | 10 | 10 |
| BW | 26.48  ±0.461 | 26.13±0.660 | 26.39±0.622 | 26.25±0.542 | 26.64±0.753 | 26.21±0.717 |
| HR | 511±3.421 | 508±3.766 | 505±3.801 | 502±3.587 | 499±3.963 | 503±4.015 |
| EF (%) | 57.670±1.391 | 30.190±1.702 | 30.120±2.113 | 39.630±1.527 | 40.550±1.762 | 49.340±1.648 |
| FS (%) | 29.760±0.909 | 13.980±0.861 | 14.050±1.070 | 18.990±0.877 | 19.420±0.997 | 24.600±0.976 |
| LVID_d_ | 3.660±0.135 | 3.956±0.102 | 4.210±0.070 | 3.958±0.202 | 3.734±0.144 | 3.869±0.074 |
| LVID_s_ | 2.572±0.106 | 3.403±0.094 | 3.615±0.081 | 3.200±0.157 | 3.005±0.112 | 2.919±0.072 |
| IVS_d_ | 0.815±0.055 | 0.739±0.055 | 0.745±0.046 | 0.899±0.054 | 0.771±0.125 | 0.832±0.153 |
| IVS_s_ | 1.183±0.048 | 0.908±0.049 | 0.855±0.070 | 1.117±0.114 | 0.978±0.058 | 1.092±0.051 |
| LVPW_d_ | 0.847±0.060 | 0.712±0.041 | 0.696±0.026 | 0.767±0.067 | 0.807±0.071 | 0.809±0.049 |
| LVPW_s_ | 1.138±0.051 | 0.760±0.069 | 0.815±0.040 | 0.923±0.078 | 0.919±0.069 | 1.068±0.068 |

Data are expressed as mean ± SEM. Data was analyzed using one-way ANOVA followed by Bonferroni post hoc analysis. BW, body weight; HR, heart rate; EF, ejection fraction; FS, fractional shortening; LVID_d_, left ventricular internal diameter at end-diastole; LVID_s_, left ventricular internal diameter at end-systole; IVS_d_, interventricular septum thickness at end-diastole; IVS_s_, interventricular septum thickness at end-systole; LVPW_d_, left ventricular posterior wall at end-diastole; LVPW_s_, left ventricular posterior wall at end-systole.
